# Biofilm Microenvironment Activated Antibiotic Adjuvant for Implant‐Associated Infections by Systematic Iron Metabolism Interference

**DOI:** 10.1002/advs.202400862

**Published:** 2024-02-26

**Authors:** Jianing Ding, Xin Wang, Wei Liu, Cheng Ding, Jianrong Wu, Renke He, Xianlong Zhang

**Affiliations:** ^1^ Department of Orthopaedics Shanghai Sixth People's Hospital Affiliated to Shanghai Jiao Tong University School of Medicine Shanghai 200233 P. R. China; ^2^ Shanghai Institute of Ultrasound in Medicine Shanghai Sixth People's Hospital Affiliated to Shanghai Jiao Tong University School of Medicine Shanghai 200233 P. R. China

**Keywords:** antibiotic adjuvant, biofilm, implant‐associated infection, iron metabolism interference

## Abstract

Hematoma, a risk factor of implant‐associated infections (IAIs), creates a Fe‐rich environment following implantation, which proliferates the growth of pathogenic bacteria. Fe metabolism is a major vulnerability for pathogens and is crucial for several fundamental physiological processes. Herein, a deferiprone (DFP)‐loaded layered double hydroxide (LDH)‐based nanomedicine (DFP@Ga‐LDH) that targets the Fe‐rich environments of IAIs is reported. In response to acidic changes at the infection site, DFP@Ga‐LDH systematically interferes with bacterial Fe metabolism via the substitution of Ga^3+^ and Fe scavenging by DFP. DFP@Ga‐LDH effectively reverses the Fe/Ga ratio in *Pseudomonas aeruginosa*, causing comprehensive interference in various Fe‐associated targets, including transcription and substance metabolism. In addition to its favorable antibacterial properties, DFP@Ga‐LDH functions as a nano‐adjuvant capable of delaying the emergence of antibiotic resistance. Accordingly, DFP@Ga‐LDH is loaded with a siderophore antibiotic (cefiderocol, Cefi) to achieve the antibacterial nanodrug DFP@Ga‐LDH‐Cefi. Antimicrobial and biosafety efficacies of DFP@Ga‐LDH‐Cefi are validated using ex vivo human skin and mouse IAI models. The pivotal role of the hematoma‐created Fe‐rich environment of IAIs is highlighted, and a nanoplatform that efficiently interferes with bacterial Fe metabolism is developed. The findings of the study provide promising guidance for future research on the exploration of nano‐adjuvants as antibacterial agents.

## Graphical abstract

A DFP@Ga‐LDH‐Cefi nanomedicine is designed to target the hematoma‐created Fe‐rich environments of IAIs by bacterial Fe metabolism intervention. Functioning as a nano‐adjuvant, DFP@Ga‐LDH‐Cefi realizes favorable antibacterial properties while delaying the emergence of drug‐resistant bacteria. These findings provide promising guidance for future research on the exploration of antibiotic nanoadjuvants and the complete treatment of IAIs.

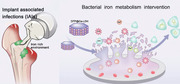

## 1 Introduction

Medical implants, particularly joint prostheses, have significantly transformed medical practice. Notably, infections originating from implanted medical devices are increasing.^[^ ^1^ ^]^ Pathogenic bacteria in IAIs tend to colonize implants as tough biofilms.^[^ ^2^ ^]^ These biofilms consist of extracellular polymeric substances, rendering them highly resistant to antibiotics.^[^ ^3^ ^]^ Accordingly, treatment of IAIs necessitates extended durations and high doses of antibiotics, thus leading to the emergence of antibiotic‐resistant bacteria.^[^ ^4^ ^]^ Presently, devising antimicrobial strategies for the comprehensive treatment of IAIs is necessary.

The intervention of the bacterial metabolism holds notable potential for achieving effective sterilization while inhibiting the emergence of drug‐resistant strains.^[^ ^5^ ^]^ Among various bacterial metabolic processes, Fe metabolism plays a pivotal role in the growth and proliferation of bacteria,^[^ ^6^ ^]^ where Fe serves as a vital cofactor for several enzymes.^[^ ^7^ ^]^ Reduction of Fe concentration by the immune system is crucial in the host‐pathogen battle.^[^ ^8^ ^]^ Moreover, inhibition of bacterial Fe acquisition could mitigate the emergence of antibiotic resistance.^[^ ^9^ ^]^

Surgical implantation invariably causes bleeding, leading to the development of a hematoma. Closed suction drainage is essential in preventing hematoma formation.^[^ ^10^ ^]^ However, after the abandonment of closed suction drainage and the widespread adoption of anticoagulants, the prevalence and severity of hematoma are on the rise.^[^ ^11^ ^]^ When the accumulation of Fe from hematoma surpasses the macrophages’ ability to process it, it significantly increases the occurrence and severity of IAIs.^[^ ^12^ ^]^ Additionally, a Fe‐rich environment plays a role in the development of antibiotic‐resistant bacteria.^[^ ^13^ ^]^ Consequently, antimicrobial strategies targeting bacterial Fe metabolism in hematoma‐created Fe‐rich environments are highly promising approaches for tackling IAIs.

Element substitution has been demonstrated to efficiently disrupt bacterial Fe metabolism. Our previous studies have revealed that Se can substitute for S in bacterial hydrogen sulfide metabolism, thereby perturbing bacterial metabolic processes.^[^ ^14^ ^]^ Ga^3+,^ as a Fe contender, competes with Fe^3+^ to bind to the vital enzymes that normally bind to Fe^3+^.^[^ ^15^ ^]^ Moreover, Ga‐containing pharmaceuticals are relatively safe as gallium nitrate has been approved by the Food and Drug Administration (FDA) for malignant hypercalcemia.^[^ ^16^ ^]^ Nevertheless, the efficacy of substituting bacterial Fe with Ga can be attenuated by excessively high Fe concentrations and limited distribution of gallium into the infected site. Fe chelators could augment the effectiveness of Ga substitution in hematoma‐created Fe‐rich environments.^[^ ^17^ ^]^ Deferiprone (DFP), an oral Fe chelator, has been approved by the FDA for Fe‐overloaded diseases such as hemochromatosis and thalassemia. By scavenging bacterial Fe, DFP could treat Gram‐negative bacterial infections.^[^ ^18^ ^]^ However, it needs an effective delivery strategy for the targeted enrichment of DFP and Ga^3+^ at the site of infection. Among nanomaterials for drug delivery, layered double hydroxides (LDHs) are ideal due to their chemical stability, biocompatibility, and pH‐dependent solubility. The lamellar structure and high surface‐to‐volume ratio of LDHs allow therapeutic agents to be intercalated.^[^ ^19^ ^]^ We hypothesized that doping LDH with Ga^3+^ is a promising DFP delivery system for therapeutic agents and gallium, thus achieving accumulation of DFP and Ga^3+^ in the infected area. This design can reduce systemic side effects of ga‐based antibacterial agents and increase the accumulation of gallium at the infection site.

**Scheme 1:**
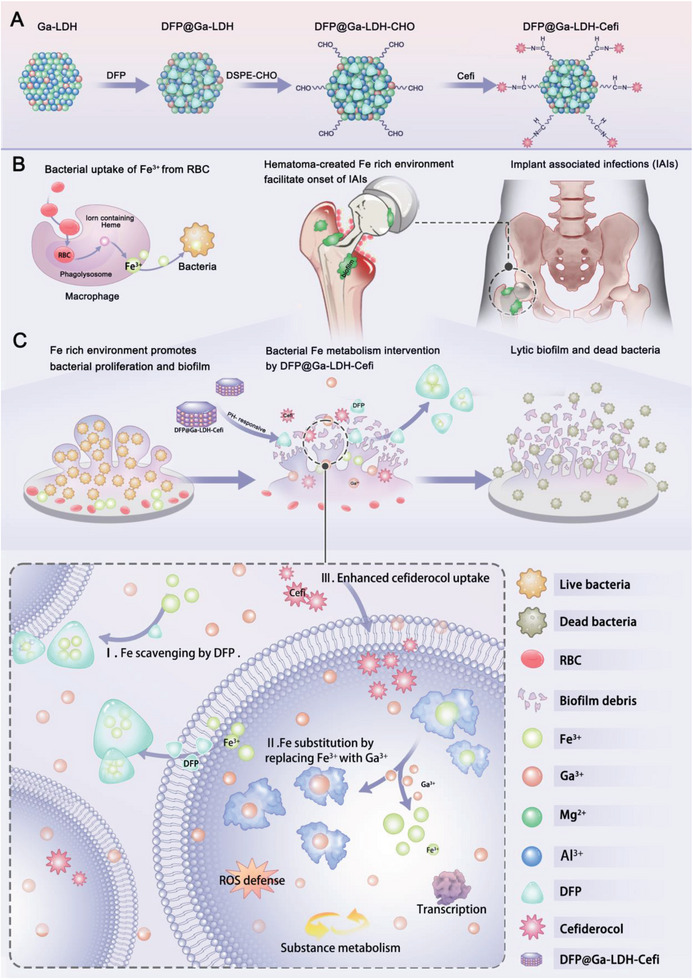
A) The synthesis process of DFP@Ga‐LDH‐Cefi. B) The hematoma formation after implantation creating a Fe‐rich environment. Macrophages phagocytose RBCs from hematomas, which results in the release of Fe and heme from hemoglobin. Heme and Fe could be absorbed by bacteria, thus facilitating bacterial proliferation and contributing to the development and severity of IAIs. C) The therapeutic mechanism of DFP@Ga‐LDH‐Cefi for eradicating IAIs by bacterial Fe metabolism intervention. DFP@Ga‐LDH‐Cefi exhibited controlled releases of Ga^3+^, DFP, and Cefi in acidic IAI environments. The synergistic action of Ga^3+^ and DFP led to effective element substitution and Fe scavenging, causing intervention in bacterial Fe‐related targets such as transcription, substance metabolism, and ROS defense.

Herein, we report a systematic Fe metabolism interference strategy based on a DFP‐loaded Ga‐doped LDH (Ga‐LDH) nanomedicine (DFP@Ga‐LDH) for the treatment of IAIs (**Scheme** **1**). In response to the acidic microenvironment at the infection site, DFP@Ga‐LDH is dissociated to release Ga^3+^ and DFP, thereby chelating Fe and reversing the intracellular Fe and Ga levels. Our experiments indicated that 200 µg/mL of DFP@Ga‐LDH could significantly reduce *Pseudomonas (P.) aeruginosa*’s Fe content to 19.34%. Multiple Fe‐related targets in *P. aeruginosa*, such as siderophore synthesis and transcription, were intervened. Additionally, DFP@Ga‐LDH, as a powerful antibiotic adjuvant, demonstrated considerable synergistic antibacterial activity with the siderophore antibiotic Cefi for preventing resistant bacteria from evolving. Thus, we subsequently introduced Cefi into DFP@Ga‐LDH to obtain DFP@Ga‐LDH‐Cefi. Through in vitro, in vivo, and ex vivo evaluations, it has been demonstrated that DFP@Ga‐LDH‐Cefi possesses superior biocompatibility and antimicrobial efficacy. This work highlighted the pivotal role of the hematoma‐created Fe‐rich environment of IAIs and developed a nanoplatform that efficiently interfered with bacterial Fe metabolism. In addition to its favorable antibacterial properties, DFP@Ga‐LDH functions as a nano‐adjuvant capable of combating antibiotic resistance, which presents significant therapeutic potential in IAIs treatments.

## 2 Results and Discussion

### 2.1 Synthesis and Characterization of DFP@Ga‐LDH‐Cefi

**Figure 1:**
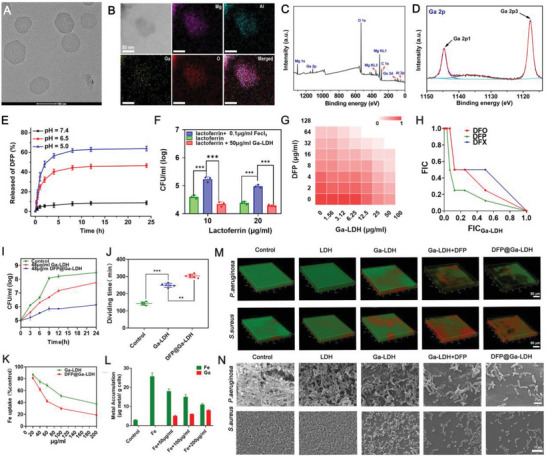
Characterization and antibacterial efficacy of Ga‐LDH nanosheets and DFP@Ga‐LDH‐Cefi. A) TEM image of Ga‐LDH nanosheets. B) HAADF‐STEM and elemental mapping images of Mg, Al, Ga, and O in Ga‐LDH nanosheets. XPS data showing the C) survey and D) Ga 2p spectra. E) In vitro cumulative release of DFP from DFP@Ga‐LDH‐Cefi at different pH values. F) The growth‐inhibitory effects of Ga‐LDH after being supplemented with Fe (FeCl_3_). G) Checkerboard microdilution assay for the combination between DFP and Ga‐LDH against *P. aeruginosa*. H) Isobologram of the combination between Ga‐LDH and DFP/DFO/DFX. I) The growth inhibition of *P. aeruginosa* after treatment with Ga‐LDH and DFP@Ga‐LDH. J) Division times of the attached bacteria with incubation of Ga‐LDH and DFP@Ga‐LDH. K) Fe uptake of *P. aeruginosa* after treatment with Ga‐LDH and DFP@Ga‐LDH. L) The Fe/Ga ratio of *P. aeruginosa* after incubation with DFP@Ga‐LDH. M) 3D reconstructions of fluorescence‐labeled *P. aeruginosa* and *S. aureus* biofilms (Scale bars: 50 µm). N) High‐resolution SEM images of *P. aeruginosa* and *S. aureus* biofilms (Scale bars: 1 µm.) (^**^ *p* < 0.01 and ^***^ *p* < 0.001; Data are expressed in mean ± SD).

Schematics of the preparation and antibacterial therapeutic performance of DFP@Ga‐LDH‐Cefi are shown in Scheme 1. Ga‐doped LDH nanosheets were fabricated via a “bottom‐up” approach, in which Ga^3+^ was doped in LDH host layers (mass content: 11.2%) to realize the relative replacement of Fe^3+^ by Ga^3+^ in the biofilm microenvironment. Specific surface areas of Ga‐LDH nanosheets were calculated to be 267.4 m/g (Table S1, Supporting Information), offering the possibility of efficient loading of small molecules into these nanosheets. In this study, Ga‐LDH nanosheets were loaded with DFP to acquire DFP@Ga‐LDH. Transmission electron microscopy (TEM) image revealed well‐dispersed sheet structures of Ga‐LDH and DFP@Ga‐LDH nanosheets (**Figure** **1**A; Figure S1, Supporting Information). Elemental mapping demonstrated uniform distributions of Mg, Al, Ga, and O in these nanosheets, confirming the successful formation of Ga‐LDH nanosheets (Figure 1B). Moreover, X‐ray photoelectron spectroscopy (XPS) profiles indicated two Al^3+^ satellite peaks at 74.5 eV and a Mg^2+^ satellite peak at 1303.6 eV, indexed to Al^3+^ and Mg^2+^ in LDH, respectively (Figure S2, Supporting Information). Furthermore, the characteristic peaks of Ga 2p at 1117.8 and 1144.6 eV were assigned to 2p3 and 2p1 of Ga^3+^ (Figure 1C,D), respectively, suggesting successful doping of Ga^3+^ into the LDH laminate. Hydrodynamic diameters of Ga‐LDH nanosheets were 113.3 ± 3.5 nm, and they increased to 119.6 ± 2.8 nm after DFP loading. Zeta potentials of the Ga‐LDH nanosheets decreased from 39.7 to 18.7 mV after DFP loading (DFP@Ga‐LDH), which was ascribed to the loading of negatively charged DFP into these nanosheets (Table S1, Supporting Information).

Moreover, Cefi was conjugated to DFP@Ga‐LDH via a pH‐sensitive imine covalent bond, thus yielding the representative antibacterial nanodrug DFP@Ga‐LDH‐Cefi. The conjugation of Cefi to DFP@Ga‐LDH exhibited no substantial influence on the hydrodynamic diameter of DFP@Ga‐LDH (Table S1, Supporting Information). Brunauer–Emmett–Teller (BET) surface areas of the Ga‐LDH nanosheets considerably decreased after DFP loading and subsequent Cefi conjugation. Changes in the zeta potentials of the Ga‐LDH nanosheets after DFP loading and subsequent Cefi conjugation further verified the effective preparation of DFP@Ga‐LDH‐Cefi. Furthermore, the rate of DFP and Cefi was evaluated by thermogravimetric (TG) analysis. The results indicate that the weight losses of Ga‐LDH, DFP@Ga‐LDH, DFP@Ga‐LDH‐CHO, and DFP@Ga‐LDH‐Cefi were 37.33%, 52.45%, 60.82%, and 66.12%, respectively (Figure S3, Supporting Information). Thus, the rates of DFP and Cefi in the DFP@Ga‐LDH‐Cefi were calculated to be 15.12% and 5.3%, respectively.

LDH‐based nanomaterials with pH‐responsive biodegradabilities have been extensively investigated as pH‐responsive nanocarriers for drug delivery. Therefore, we examined the DFP release behavior of DFP@Ga‐LDH‐Cefi at different pH values. At pH 7.4, the amount of DFP released from DFP@Ga‐LDH‐Cefi was less than 10% after 24 h, and almost no Ga^3+^ was released from DFP@Ga‐LDH‐Cefi (Figure 1E; Figure S4, Supporting Information), suggesting high stability of DFP@Ga‐LDH‐Cefi in a normal physiological environment. In contrast, at pH 6.5 and 5.0, the amounts of released DFP increased to 44.5% and 68.9% within 8 h. Additionally, the release pattern of Ga^3+^ was highly pH‐dependent, which was attributed to the dissolution of the LDH carrier under acidic conditions. These results further confirmed the spatial specificity of DFP@Ga‐LDH‐Cefi for DFP and Ga^3+^ released into the bacterial microenvironment.

### 2.2 Significant Antibiofilm Efficacy of DFP@Ga‐LDH Due to Fe Chelation and Element Substitution

Ga‐LDH nanosheets exhibit considerable antibacterial effects, as evidenced by the MIC against *P. aeruginosa* is 96 µg mL^−1^. Bacteriostatic assay further demonstrated that the Ga‐LDH nanosheets effectively inhibited the growth of PAO1 in a dose‐dependent manner (Figure S5A, Supporting Information). The 24‐h bactericidal assay indicated that 250 µg mL^−1^ Ga‐LDH nanosheets eradicated 99% of stationary‐phase bacteria (Figure S5B, Supporting Information). As the antibacterial properties of Ga^3+^ relied on its ability to substitute bacterial Fe^3+^, our study aimed to investigate the impact of Fe concentration on the antibacterial efficacies of Ga‐LDH nanosheets. FeCl_3_ and lactoferrin, a natural chelating agent, were separately introduced into Ga‐LDH nanosheets to modify their Fe concentrations. Results revealed a notable decline in the antibacterial properties of the Ga‐LDH nanosheets upon the addition of FeCl_3_ (Figure S5C, Supporting Information). Contrarily, lactoferrin significantly enhanced the antibacterial properties of the Ga‐LDH nanosheets (Figure 1F). These findings demonstrate that the antibacterial efficiencies of Ga‐LDH nanosheets are influenced by Fe concentration, suggesting the potential of utilizing Fe chelators to enhance the antibacterial efficacies of Ga‐LDH nanosheets. Defersirox (DFX), deferoxamine (DFO), and DFP, which are FDA‐approved Fe chelators, are extensively used to treat Fe overload diseases,^[^ ^20^ ^]^ and possess inherent antimicrobial activities.^[^ ^21^ ^]^ Checkerboard microdilution assays were performed to analyze the synergistic effects of Ga‐LDH nanosheets and Fe chelators, which revealed that the combination of Ga‐LDH nanosheets and DFP exhibited the highest level of antibacterial activity (Figure 1G,H; Figure S6, Supporting Information). Therefore, herein, a nanomaterial platform, DFP@Ga‐LDH, was constructed by loading Ga‐LDH nanosheets with DFP. DFP@Ga‐LDH demonstrated a MIC of 48 µg/mL against *P. aeruginosa*. Moreover, the bactericidal assay verified the significant antibacterial activity of DFP@Ga‐LDH as compared to those of Ga‐LDH nanosheets (Figure 1I; Figure S5D, Supporting Information). These results imply superior antibacterial efficacy of DFP@Ga‐LDH to those of Ga‐LDH nanosheets.

Interventions in bacterial Fe metabolism can impede bacterial proliferation.^[^ ^22^ ^]^ Notably, both DFP@Ga‐LDH and Ga‐LDH nanosheets demonstrated inhibitory effects on the growth of *P. aeruginosa*, and DFP@Ga‐LDH exhibited more considerable growth inhibition (Figure 1J). Speculating that DFP@Ga‐LDH intervened in Fe metabolism via Fe scavenging and element substitution, we conducted the following experiments. ICP‐MS data demonstrated concentration‐dependent inhibitory effects of both Ga‐LDH nanosheets and DFP@Ga‐LDH on bacterial Fe content. Remarkably, DFP@Ga‐LDH exhibited more prominent inhibitory effects on bacterial Fe content than those of Ga‐LDH nanosheets (Figure 1K). Further investigations were performed to assess Fe substitution by DFP@Ga‐LDH. After co‐cultivation with DFP@GaLDH and *P.aeruginosa*, DFP@Ga‐LDH did not adhere to bacterial cells(Figure S7, Supporting Information).With a gradual increase in the concentration of DFP@Ga‐LDH, the bacterial Fe load progressively reduced, whereas the Ga load concomitantly increased (Figure 1L). These findings validate the effectiveness of chelating Fe^3+^ and substituting Fe^3+^ with Ga^3+^ using DFP@Ga‐LDH.

Inhibiting the adhesion of bacteria to biomaterials can effectively suppress biofilm formation, which is a critical aspect of IAIs treatment. Effective interventions in bacterial Fe metabolism can impede bacterial adhesion.^[^ ^23^ ^]^ Our results revealed that DFP@Ga‐LDH effectively inhibited bacterial adhesion and biofilm formation(Figures S8–S10, Supporting Information). Subsequently, scanning electron microscopy (SEM) and confocal laser scanning microscopy (CLSM) were performed to analyze the effects of DFP@Ga‐LDH on biofilm formation. After treatment with DFP@Ga‐LDH, the biofilm was significantly disrupted with thin and dispersed features (Figure 1M). In contrast, in the cases of the control and LDH groups, the 3D structures of biofilms remained intact and the bacteria were highly active. SEM revealed substantial bacterial densities and well‐preserved bacterial morphologies for both the control and LDH groups. Nevertheless, the majority of *P. aeruginosa* and *Staphylococcus (S.) aureus* within the biofilm demonstrated shriveling, distortion, or complete lysis after incubation with DFP@Ga‐LDH (Figure 1N). Antibiofilm efficacy of DFP@Ga‐LDH was evaluated against both *P. aeruginosa* and *S. aureus*, and a standard crystal violet assay of the biofilm biomass indicated that DFP@Ga‐LDH efficiently inhibited biofilm formation and eradicated the preformed biofilm (Figures S11, and S12, Supporting Information).

### 2.3 Simultaneous Intervention of DFP@Ga‐LDH in Multiple Fe‐Related Targets Within *P. aeruginosa*

**Figure 2:**
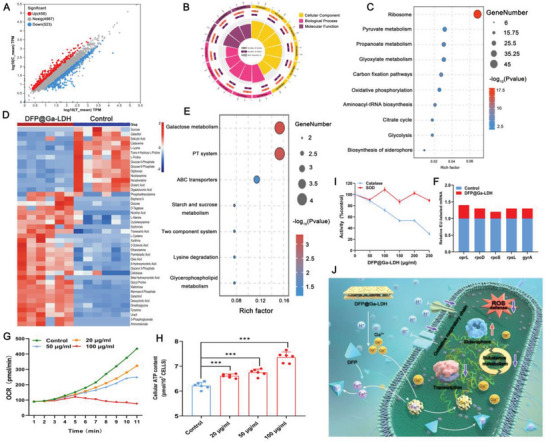
DFP@Ga‐LDH alters multiple Fe‐dependent targets. A) Volcano plots of differentially expressed genes (gray: genes that are not significantly different; red: upregulated genes; and blue: downregulated genes). B) GO enrichment analysis of transcriptomics. C) KEGG enrichment analysis of transcriptomics. D) Heat map for the relative abundance of metabolites in *P. aeruginosa* upon DFP@Ga‐LDH treatment. E) KEGG enrichment analysis of metabolomics. F) Analysis of mRNA levels of *P. aeruginosa* treated with or without DFP@Ga‐LDH. G) Real‐time changes in the oxygen consumption rate of *P. aeruginosa* after treatment with DFP@Ga‐LDH. H) ATP content of *P. aeruginosa* under DFP@Ga‐LDH treatment. I) The activity of Catalase and SOD after treatment with DFP@Ga‐LDH. J) Schematic of the Fe‐related targets of DFP@Ga‐LDH. (^*^ *p* < 0.05, ^**^ *p* < 0.01, and ^***^ *p* < 0.001; ns means no significance. Data are expressed in mean ± SD).

The abovementioned findings confirm the effectiveness of DFP@Ga‐LDH in efficiently chelating Fe^3+^ and substituting Fe^3+^ with Ga^3+^. In the following investigations, we aimed to elucidate the intervention of DFP@Ga‐LDH in precise Fe‐related targets. At first, to investigate the global nature of *P. aeruginosa* following treatment with DFP@Ga‐LDH, transcriptomic analysis was conducted(Figure S13A‐C, Supporting Information). Compared to the control group, the DFP@Ga‐LDH group demonstrated significant differential expression, with 458 upregulated and 523 downregulated genes (**Figure** **2**A). Gene Ontology (GO) and Kyoto Encyclopedia of Genes and Genomes (KEGG) analyses, which are widely utilized functional enrichment analyses in transcriptomics, were employed to predict the potential targets intervened by DFP@Ga‐LDH. Notably, GO analysis revealed the substantial effects of DFP@Ga‐LDH on crucial biological processes, for example, siderophore biosynthesis, ribosomes, metabolism, and electron transport chain, in bacteria (Figure 2B). Furthermore, KEGG analysis indicated the significant impacts of DFP@Ga‐LDH on important pathways, including ribosomes, citrate cycle, siderophore biosynthesis, and oxidative phosphorylation, in bacteria (Figure 2C). These results implied that siderophore synthesis, transcription, substance metabolism, and oxidative phosphorylation were potential targets for DFP@Ga‐LDH.

Alterations in substance metabolism can influence the efficiencies of antimicrobial agents.^[^ ^24^ ^]^ Metabolomics was employed to examine alterations in the metabolite abundance of *P. aeruginosa* induced by DFP@Ga‐LDH(Figure 13D‐E, Supporting Information). After a 4‐h intervention, the DFP@Ga‐LDH‐treated group exhibited a significant increase in the abundance of diverse metabolites (Figure 2D), accompanied by a concomitant elevation in intracellular adenosine triphosphate (ATP) levels (Figure 2H). The observed accumulation of multiple metabolites signified that metabolic rate and energy utilization decreased after DFP@Ga‐LDH treatment. Pathway enrichment analysis confirmed the substantial suppression of substance metabolism by DFP@Ga‐LDH (Figure 2E).

Ribonucleic acid (RNA) polymerase, a Fe‐containing enzyme, is possibly responsible for the observed transcriptional repression.^[^ ^25^ ^]^ The reduced expression of housekeeping genes in *P. aeruginosa* confirmed the transcriptional inhibition caused by DFP@Ga‐LDH, as shown in Figure 2F. This target is the same as that of the canonical antibiotic rifampicin. Oxygen consumption rate (OCR) was used as an indicator of electron transport chain activity because oxygen is the terminal electron acceptor. DFP@Ga‐LDH supplementation progressively decreased the OCR of *P. aeruginosa* (Figure 2G), indicating inhibition of bacterial respiratory activity. Bacterial reactive oxygen species (ROS)‐scavenging enzymes, such as Fe‐superoxide dismutase and catalases, play critical roles as antioxidant enzymes. The addition of DFP@Ga‐LDH resulted in a 70% decrease in bacterial catalase activity, however, no significant alteration was noticed in bacterial superoxide dismutase activity (Figure 2I). These findings suggest that DFP@Ga‐LDH can weaken the bacterial ROS defense mechanism, rendering the bacterium more susceptible to oxidative stress, and various antibiotics, clinical oxidants, and photodynamic therapy demonstrate the same mechanism.^[^ ^26^ ^]^

According to the preceding discussion, a schematic depicts the observed outcomes, which reveals that DFP@Ga‐LDH exerts a discernible impact on multiple Fe‐related processes, including transcription, substance metabolism, oxidative phosphorylation, siderophore synthesis, and defense against ROS, in *P. aeruginosa* (Figure 2J).

### 2.4 DFP@Ga‐LDH: A Nano‐Adjuvant with Considerable Antiresistant Activity

**Figure 3:**
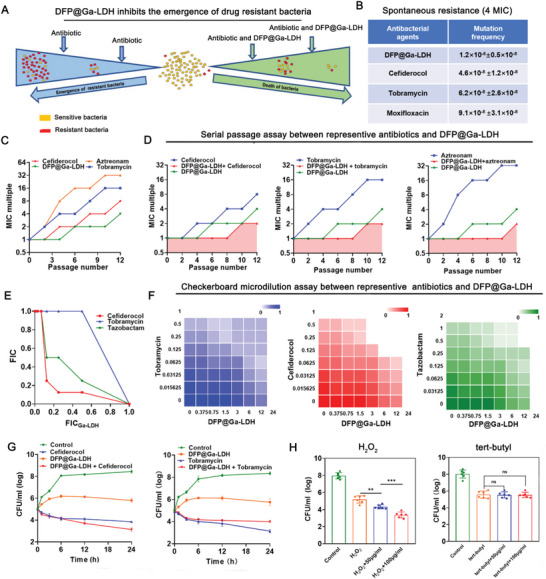
DFP@Ga‐LDH functions as a nano adjuvant capable of delaying the emergence of drug resistance and sensitizing antibacterial antibiotics. A) Schematic illustrating the mechanism of DFP@Ga‐LDH as a resistance‐modifying nano‐adjuvant. B) Spontaneous resistance of DFP@Ga‐LDH and classic antibiotics. C,D) Resistance acquisition curves with the subinhibitory concentration of representative antibiotics (C) or combination of representative antibiotics and DFP@Ga‐LDH (D) against *P. aeruginosa*. (MIC test was performed every two passages.) E) Isobolograms of the combinations between antibiotics and DFP@Ga‐LDH. F) Checkerboard microdilution assay for the combinations between DFP@Ga‐LDH and representative antibiotics. G) The synergistic effects of DFP@Ga‐LDH in combination with cefiderocol and tobramycin. H) Effect of DFP@Ga‐LDH on the sensitivity of *P. aeruginosa* to H_2_O_2_ and tert‐butyl. (^***^ *p* < 0.001; ns means no significance).

Prolonged treatment of IAIs facilitates the emergence of drug‐resistant bacteria, which commonly lead to treatment failure.^[^ ^27^ ^]^ The aforementioned findings provide compelling evidence that DFP@Ga‐LDH simultaneously targets multiple Fe‐related processes. This mode of intervention targeting bacterial Fe metabolism holds the potential in delaying the progression of antibiotic resistance.^[^ ^28^ ^]^ Subsequent experiments demonstrated the ability of DFP@Ga‐LDH to effectively suppress the emergence of drug‐resistant bacteria (**Figure** **3**A). Spontaneous resistance refers to the ability of bacteria to naturally develop drug resistance. Our findings revealed that ≈1 in 80 million *P. aeruginosa* spontaneously developed resistance to DFP@Ga‐LDH, which was lower than the resistance frequencies observed for several tested classical antibiotics (Figure 3B). Moreover, serial passage offers valuable insights into the dynamics of drug resistance development.^[^ ^29^ ^]^ Notably, after serial passage, the development of resistance to DFP@Ga‐LDH was substantially slower than those in the cases of conventional antibiotics (Figure 3C). The combination of DFP@Ga‐LDH with Cefi, tazobactam, and tobramycin significantly reduced the prevalence of resistant bacteria (Figure 3D; Figure S15, Supporting Information). Next, a checkerboard microdilution assay was used to investigate the combined antibacterial effects of DFP@Ga‐LDH and the tested antibiotics. The combination of DFP@Ga‐LDH with Cefi and tazobactam exhibited synergistic effects, and this observation was supported by the time‐kill curves. However, DFP@Ga‐LDH demonstrated antagonistic effects against tobramycin (Figure 3E–G; Figure S16, Supporting Information). Oxidizing agents are extensively employed in skin disinfection and debridement surgeries.^[^ ^30^ ^]^ In accordance with the previous findings substantiating the inhibitory effect of DFP@Ga‐LDH on catalase activity, hydrogen peroxide (H_2_O_2_) exhibited high sensitivity against *P. aeruginosa* in the presence of DFP@Ga‐LDH. Contrarily, DFP@Ga‐LDH did not demonstrate high sensitivity to tert‐butyl (Figure 3H).

In conclusion, DFP@Ga‐LDH as an antibiotic adjuvant suppressed the emergence of drug‐resistant bacteria and enhanced the bactericidal efficiencies of conventional antibiotics and clinical oxidants.

### 2.5 Validation of Antimicrobial Efficacy and Biosafety of DFP@Ga‐LDH‐Cefi

Among the antibiotics tested herein, DFP@Ga‐LDH exhibited a prominent synergistic effect with the novel siderophore cephalosporin Cefi. This synergistic effect can be attributed to the intervention in bacterial Fe metabolism by DFP@Ga‐LDH, which enhanced siderophore synthesis (Figure S14, Supporting Information). Utilizing the considerable drug‐loading capacities of LDH materials, we successfully conjugated Cefi to DFP@Ga‐LDH, resulting in DFP@Ga‐LDH‐Cefi.

**Figure 4:**
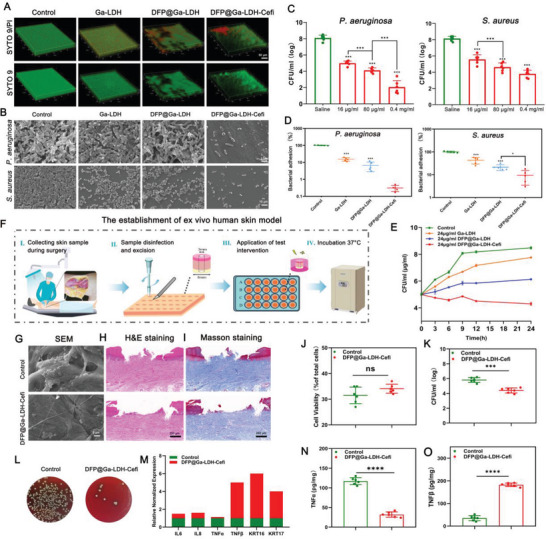
Validation of the antimicrobial efficacy and biosafety of DFP@Ga‐LDH‐Cefi. A) Representative images of 3D reconstructions of *P. aeruginosa* biofilm stained with SYTO 9/PI (Scale bar: 50 µm). B) Representative SEM images of *P. aeruginosa* and *S. aureus* biofilms. C) 24‐h killing assays of *P. aeruginosa* and *S. aureus* conducted using DFP@Ga‐LDH‐Cefi. D) Number of adherent viable bacteria after incubation with DFP@Ga‐LDH‐Cefi. E) DFP@Ga‐LDH‐Cefi inhibits *P. aeruginosa* growth in a concentration‐dependent manner. F) Schematics for the preparation of an ex vivo human skin model and experimental process. G) SEM images of wound bed after various treatments (Scale bar: 5 µm). H,I) H&E staining (H) and Masson staining (I) of the wound bed after several treatments (Scale bar: 250 µm). J) Cell viability of skin tissue after treatment with DFP@Ga‐LDH‐Cefi. K) Quantification of viable bacteria. L) Representative images of SPM from *S. aureus* after DFP@Ga‐LDH‐Cefi treatment. M) Gene expression of pro‐inflammatory and pro‐reparative cytokines. N) Quantification of TNF‐α and O) TGF‐β proteins in culture media. (^*^ *p* < 0.05, ^**^ *p* < 0.01, ^***^ *p* < 0.001, and ^****^ *p* < 0.0001; ns means no significance. Data are expressed in mean ± SD).

In vitro experiments demonstrated that DFP@Ga‐LDH‐Cefi outperformed DFP@Ga‐LDH in combating both planktonic bacteria and biofilms (**Figure** **4**A–C,E; Figures S17–S19, Supporting Information). Bacterial adhesion is an initial and crucial step in biofilm formation, and the effective inhibition of bacterial adhesion is paramount for preventing IAIs.^[^ ^31^ ^]^ DFP@Ga‐LDH‐Cefi substantially decreased the adhesion of both *P. aeruginosa* and *S. aureus* to the biomaterials (Figure 4D). An ex vivo human skin model was established to evaluate the antimicrobial efficacy and biocompatibility of DFP@Ga‐LDH‐Cefi.^[^ ^32^ ^]^ The experimental procedure is outlined in Figure 4F. The DFP@Ga‐LDH‐Cefi group demonstrated a comparable percentage of viable cells to the control group, indicating the biosafety of DFP@Ga‐LDH‐Cefi (Figure 4J). SEM images clearly indicated that treatment with DFP@Ga‐LDH‐Cefi effectively suppressed biofilm formation, as evidenced by the reduced presence of *S. aureus* and diminished extracellular matrix as compared to the case of the control group (Figure 4G). After culturing the excised skin wounds under aseptic conditions, hematoxylin and eosin (H&E) and Masson stainings were conducted, and results revealed that the control group exhibited no signs of wound re‐epithelialization due to severe *S. aureus* infection. In contrast, the DFP@Ga‐LDH‐Cefi group, in which *S. aureus* infection was effectively suppressed, demonstrated noticeable wound re‐epithelialization (Figure 4H,I). The antibacterial efficacy of DFP@Ga‐LDH‐Cefi was confirmed using the plate counting method, which involved the detection of viable bacteria in wound tissue homogenates. Results indicated that the bacterial load in the DFP@Ga‐LDH‐Cefi group was significantly low as compared to that in the control group (Figure 4K,L). Expression levels of the inflammatory factors in skin tissue serve as indicators of the severity of bacterial infection. Following treatment with DFP@Ga‐LDH‐Cefi, the skin tissue exhibited substantially reduced expression levels of tumor necrosis factor‐α (TNF‐α), interleukin (IL)−6, and IL‐8 as compared to those in the case of the control group. This suggests that DFP@Ga‐LDH‐Cefi effectively inhibits bacterial infection in the skin tissue. Healing of infected wounds reflects the efficacy of the drug in controlling skin infections. Notably, the expression levels of transforming growth factor‐beta (TGF‐β), keratin (Krt)17, and Krt16 in the DFP@Ga‐LDH‐Cefi group were significantly high, further highlighting the positive impact of DFP@Ga‐LDH‐Cefi on wound healing and infection control (Figure 4M–O). The preceding discussion indicates outstanding biocompatibility and antibacterial properties of DFP@Ga‐LDH‐Cefi in the ex vivo human skin model. To ensure the biocompatibility of DFP@Ga‐LDH‐Cefi for further applications, the toxicities of DFP@Ga‐LDH and DFP@Ga‐LDH‐Cefi were systematically assessed.

Immune cells, especially macrophages, could take up gallium. These facts raise concern that DFP@Ga‐LDH could negatively affect the efficiency of antimicrobial immunity. Our findings indicate that the antimicrobial immunity was not adversely impacted by DFP@Ga‐LDH. The viability of human monocyte‐derived macrophages (HMDM) was not reduced by DFP@Ga‐LDH treatment (Figure S20A, Supporting Information). We also investigated the impact of continuous exposure to DFP@Ga‐LDH (1 mg mL^−1^) for 24 h on macrophage antimicrobial activity. Our findings indicate that this treatment sightly enhances the ability of macrophage in *P. aeruginosa* killing (Figure S20B, Supporting Information). In addition, DFP@Ga‐LDH had a modest impact on M1 polarization (Figure S20C, Supporting Information). In vitro cytotoxicity tests and in vivo toxicity evaluations revealed no substantial toxicities of DFP@Ga‐LDH and DFP@Ga‐LDH‐Cefi (Figures S21–S27, Supporting Information).

### 2.6 In Vivo Anti‐Infection Evaluations of DFP@Ga‐LDH‐Cefi Using a Mouse IAI Model

**Figure 5:**
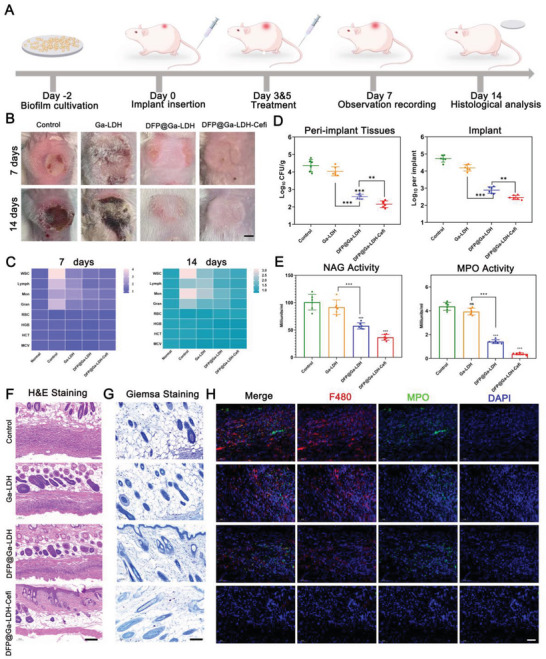
In vivo anti‐infection evaluations of nanodrugs using the mouse IAI model. A) Timeline illustration of the mouse IAI model. B) Representative images of the local infection site at different time points (Scale bar: 25 mm). C) Routine blood test at 7 and 14 days. D) Quantification of viable bacteria in peri‐implant soft tissues and implants. E) Quantification of the inflammatory cell biomarkers in peri‐implant soft tissues. F) Representative H&E staining images of the histologic sections of wounds in various groups at 14 days (Scale bar: 200 µm). G) Giemsa staining of peri‐implant soft tissues visually indicated the matched bacterial load (Scale bar: 200 µm). H) Representative images of the immunofluorescence‐stained sections of peri‐implant soft tissues presenting different functional cells (Scale bar: 100 µm, Green: MPO for neutrophils, Red: F480 for macrophages, and Blue: DAPI for nuclei) (^**^ *p* < 0.01 and ^***^ *p* < 0.001. Data are expressed in mean ± SD).

Following implantation, the Fe concentration around the implant elevates to 141.8 µg g^−1^ tissue, while in normal tissues it is 48.3 µg/g tissue. This indicates the existence of a hematoma‐created Fe‐rich environment (Figure S28, Supporting Information). The abovementioned experiments demonstrated the excellent antibacterial ability of DFP@Ga‐LDH‐Cefi, which prompted us to further investigate the antibiofilm efficacy of DFP@Ga‐LDH‐Cefi in a mouse model of IAIs. A schematic illustrating the development of the mouse IAIs model and corresponding treatment is depicted in **Figure** **5**A. On day −2, sterile polyetheretherketone (PEEK) sheets were immersed in a suspension of *P. aeruginosa* to facilitate biofilm synthesis. On day 0, the PEEK sheets coated with biofilms were implanted into all mice and then randomly divided into four experimental groups: control, Ga‐LDH, DFP@Ga‐LDH, and DFP@Ga‐LDH‐Cefi groups. Different types of nanomedicines were administered on days 0, 3, and 5 for treatment. After sacrificing the mice, tissue samples were acquired from the infection site for histological and quantitative analyses. On 7 and 14 days after PEEK sheet implantation, macroscopic images of the skin tissue with subcutaneous implants were obtained (Figure 5B). In the control and Ga‐LDH groups, considerable swelling and suppuration were observed, indicating inflammatory responses at the abscess sites. In contrast, the DFP@Ga‐LDH group exhibited minimal swelling of the scabby skin tissue. Notably, the DFP@Ga‐LDH‐cefi group demonstrated no swelling, suppuration, or wound festering, implying its superior antibiofilm activity. Blood tests were performed to analyze the inflammatory markers in the mouse blood (Figure 5C). Compared with the normal group, the control group exhibited elevated levels of white blood cells, lymphocytes, monocytes, and neutrophil granulocytes. Nevertheless, the DFP@Ga‐LDH‐Cefi group demonstrated slightly elevated levels of these cells, indicating alleviation of the systemic inflammatory response. Bacterial loads of PEEK plates and peripheral tissues after the sacrifice of mice were evaluated by estimating the number of colonies of viable bacteria. As expected, the numbers of bacterial colonies in the DFP@Ga‐LDH and DFP@Ga‐LDH‐Cefi groups were substantially lower than those in the other two groups (Figure 5D). To assess the involvement of neutrophils in inflammation development tissue repair and inflammatory recovery, the two inflammatory biomarkers myeloperoxidase (MPO) and n‐acetylglucosaminidase (NAG) were quantified in the tissue, respectively. Results revealed that the DFP@Ga‐LDH‐Cefi group exhibited the highest activities of macrophages, indicating the presence of tissue repair processes, and minimal inflammatory reactions were observed in this group (Figure 5E). The H&E staining revealed significant neutrophil infiltration in the tissue surrounding the implant in the control group, indicating a pronounced inflammatory response induced by bacterial infection. However, this inflammatory response was effectively inhibited by the treatment of DFP@Ga‐LDH‐Cefi (Figure 5F). Giemsa staining revealed the presence of bacteria in tissues. In the DFP@Ga‐LDH‐Cefi group, the stained bacteria were significantly fewer than those in the other groups (Figure 5G). In the DFP@Ga‐LDH‐Cefi group, the peri‐implant soft tissue demonstrated complete healing without infiltration of macrophages and neutrophils, as evidenced by tissue immunofluorescence analysis (Figure 5H). Overall, our findings confirmed that DFP@Ga‐LDH and DFP@Ga‐LDH‐Cefi effectively alleviated IAIs.

## 3 Conclusion

In summary, herein, we successfully prepared DFP@Ga‐LDH and its Cefi‐loaded counterpart, DFP@Ga‐LDH‐Cefi. DFP@Ga‐LDH‐Cefi exhibited controlled releases of Ga^3+^, DFP, and Cefi in acidic IAI environments. The synergistic action of Ga^3+^ and DFP led to effective Fe substitution and Fe scavenging, causing intervention in bacterial Fe‐related targets such as transcription and substance metabolism. Moreover, the considerable antimicrobial activity of DFP@Ga‐LDH‐Cefi along with its ability to counteract drug resistance renders it highly promising for the treatment of IAIs. We anticipate that the investigation of bacterial Fe metabolism intervention in the Fe‐rich environments around implants along with the conceptualization of pharmaceutical design involving element chelation and substitution will serve as a source of inspiration in the fields of pharmaceutical sciences, materials engineering, and nanomedicine.

## 4 Experimental Section

### Clinical Samples

Skin samples were acquired from patients undergoing surgery at Shanghai Sixth People's Hospital. Only remnant clinically healthy skin that was not needed for diagnostic purposes was analyzed after obtaining written informed consent from the patients and approval from the Ethics Committee of Shanghai Sixth People's Hospital in accordance with current regulations and institutional guidelines. The approval number is 2022‐KY‐139(K).

### Synthesis of Ga‐LDH Nanosheets

Ga‐LDH nanosheets were synthesized by a bottom‐up method. Briefly, Al(NO_3_)_3_·9H_2_O (3 mmol), Mg(NO_3_)_2_·6H_2_O (6 mmol), and Gd(NO_3_)_3_·6H_2_O (0.1 mmol) were dissolved in deionized water (10 mL). Then, NaNO_3_ solution (1.5 mmol, 25% formamide) and 30 mL NaOH solution (1.5 mm) were slowly added to the resulting solution followed by stirring for 30 min. The resulting mixture was centrifuged (13 000 rpm) and washed twice with deionized water to obtain the Ga‐LDH nanosheets.

### Preparation of DFP@Ga‐LDH and DFP@Ga‐LDH‐Cefi

For surface modification, the as‐synthesized Ga‐LDH nanosheets (20 mg) were mixed with polyethylene glycol‐aldehyde (PEG‐CHO) aqueous solution (10 mL, molecular weight (MW): 3000) under vigorous stirring for 2 h. To remove excess and unbound PEG‐CHO, the resulting product Ga‐LDH‐CHO was acquired by centrifugation and washed several times with water and ethanol. For DFP loading, Ga‐LDH‐CHO dispersion was mixed with DFP solution at various DFP/Ga‐LDH‐CHO molar ratios (from 1:2 to 2:1) followed by stirring at room temperature for 12 h. The resulting product DFP@Ga‐LDH was obtained by centrifugation and then washed three times with deionized water, followed by redispersion in water for further use. Thereafter, DFP@Ga‐LDH was conjugated with Cefi via pH‐sensitive imine covalent bonds according to the previous study.^[^ ^33^ ^]^ Initially, Cefi (5 mg) was pre‐dissolved in water (10 mL) followed by the addition of 1‐ethyl‐3‐(3‐dimethylaminopropyl)carbodiimide (20 mg) and N‐hydroxysuccinimide (45 mg) under ultrasonication. After being stirred for 24 h, the mixture was dialyzed against water (cutoff MW: 3500 Da) for 3 days and redispersed in 5 mL ultrapure water. Subsequently, the as‐prepared DFP@Ga‐LDH dispersion and activated Cefi solution were mixed followed by the addition of NaOH (1 m) to adjust the pH value to 8.5 and stirring for 24 h. After centrifugation at 13 000 rpm three times, DFP@Ga‐LDH‐Cefi was achieved and redispersed for further use. Moreover, in vitro DFP release from DFP@Ga‐LDH was analyzed at varying pH values (7.4, 6.5, and 5.0) by a dialysis method. The absorbance of the released solution at 278 nm was measured. Additionally, 1 mL of another sample was acquired and examined using ICP‐OES to quantify the released Ga^3+^.

### Resistance Development by Sequential Passaging

Continuous passaging was performed in 96‐well plates, with one replicate per plate, to avoid cross‐contamination. BM2 medium was prepared using each agent at eight concentrations in twofold dilutions, and 5 µL PAO1 was introduced into each well. After overnight incubation, the highest concentration of the agent that allowed bacterial growth was recorded. A 5 µL aliquot of the culture from this well was transferred to a fresh medium containing the agent, and this process was repeated for 12 days. Control replicates were passed without this agent. A total of 12 replicates were passaged for each agent for 12 days.

### Mutation Rate Assays

In this experiment, PAO1 was grown to the mid‐log phase and then resuspended in a fresh medium. Aliquots of successive dilutions of the overnight cultures grown in LB media were placed on BM2 plates without and with different antibacterial agents at concentrations four times MIC for each agent. After 24 h of incubation, the number of colonies growing on the plates was counted. Thereafter, mutation frequency was calculated as the fraction of resistant mutant cells relative to the total number of cells.

### Ex Vivo Human Skin Model of Wound Infection

Skin samples were washed with 3xABAM and phosphate‐buffered saline (PBS) after the removal of adipose tissue. Subsequently, circular wounds were created using 3 mm diameter biopsy punch tools, and the wound and rim of the tissue were excised using 8 mm punch tools. After being washed twice with Dulbecco's modified Eagle medium (DMEM) supplemented with 10% fetal bovine serum (FBS), the skin explants were individually cultured in 1 mL culture medium, and the epithelium was maintained at the air/liquid interface. DMEM supplemented with 10% FBS was used as the culture medium. *S. aureus* was incubated at 37 °C in tryptic soy broth normalized to a concentration of 10^6^ colony‐forming units (CFUs) mL^−1^. Thereafter, the skin wounds were inoculated with 10 µL bacterial inoculum (10 000 CFUs) and various nanodrugs. The skin explants were cultured for 48 h, and the medium was changed every 12 h to facilitate biofilm formation and select adherent bacteria. After 48 h, the skin wounds were obtained for analysis.

### Bacterial Enumeration in Skin

At first, a quarter of each wound was placed in sterile PBS and homogenized on ice using a Tissue‐Tearor at maximum speed for 25 s. Then, the resulting homogenate was serially diluted in triplicate from 10^−1^ to 10^−5^. The diluted samples were placed on tryptic soy agar plates followed by incubation at 37 °C for 24 h. Colonies that formed on the plates were counted, and CFUs were quantified by back‐calculation based on the dilution factor. CFU represents the estimated number of viable bacteria in the original wound sample.

### Assessment of Tissue Viability

In this experiment, wound tissues were incubated ex vivo for 48 h and then minced using surgical scissors. The resulting tissue fragments were treated with dispase II solution at 37 °C for 30 min, followed by incubation with 2 mg mL^−1^ collagenase D under constant agitation for 3 h at 37 °C to obtain single‐cell suspensions. The resulting cell suspensions were washed with DMEM supplemented with 10% FBS, L‐glutamine (2 mm) 0.15% hydrogen carbonate, sodium pyruvate (1 mm), nonessential amino acids, and 100 µg/mL gentamycin. The cells were labeled using the LIVE/DEAD Fixable Yellow Dead Cell Stain Kit to distinguish between live and dead cells. The percentage of live cells was determined by acquiring ≈2 00 000 events from each sample using a BD Fortessa flow cytometer equipped with lasers emitting at 405, 488, 642, and 785 nm. These data provide valuable information on the viability of cells in wound tissue samples.

### Relative Messenger RNA (mRNA) Expression

The sample was crushed, and RNA was extracted according to the protocol of the Qiagen RNeasy Kit. After extraction and purification, complementary deoxyribonucleic acid (cDNA) was generated using the High‐Capacity cDNA Reverse Transcription Kit (Applied Biosystems, Foster City, CA) and SuperScript III Reverse Transcriptase Kit (Invitrogen, Carlsbad, CA). Changes in gene expression were determined using the CFZX384 Touch Real‐Time Fluorescence Quantitative PCR Detection System.

### Preparation of PEEK Sheets

Briefly, at first, PEEK was cut into circular sheets with diameters of 10 mm and thicknesses of 0.5 mm. These circular sheets were sterilized and transferred to a six‐well plate. Next, a 2 mL suspension of *P. aeruginosa* (10^4^ CFU/mL) was introduced onto the aforementioned plate. Subsequently, the plate was incubated under conditions of 5% CO_2_ and a temperature of 37 °C for a duration of 2 days in order to facilitate the growth of bacterial biofilm. The medium was refreshed daily. After 2 days, the PEEK sheets were acquired and used for the construction of a mouse IAI model.

### Mouse IAI Model

Animal experiments for this study were approved by the Animal Care and Experiment Committee of Shanghai Jiao Tong University Affiliated Sixth People's Hospital. The assigned approval/accreditation number of the laboratory/investigator is DWLL2021‐0674. Herein, 32 BALB/c mice were equally and randomly divided into the following four groups: control, Ga‐LDH, DFP@Ga‐LDH, and DFP@Ga‐LDH‐Cefi groups. A mouse IAI model was used to evaluate the in vivo antibiofilm ability of DFP@Ga‐LDH‐Cefi. Briefly, the mice were anesthetized with 1% pentobarbital sodium, and the skin on their backs was shaved, disinfected, and incised. Subsequently, the prepared PEEK sheets with biofilms were subcutaneously implanted, and the skin wounds were immediately sutured. After surgical implantation, the mice in different groups were injected with 1 mL of the corresponding solution via the tail vein (1 mL saline for the control group and 1 mL corresponding nanodrugs for the other three groups) at a dose of 2 mg/100 g body weight. The injection was repeated on days 3 and 5 after the first injection. Changes at the infection site were daily observed until the mice were sacrificed. Images of the infected sites were acquired on days 7 and 14. All mice were sacrificed on day 14.

### Statistical Analysis

In all the experiments, at least three replicates were included, and data were expressed as mean ± standard error of the mean (SEM). The differences between the two groups were analyzed by independent sample *t*‐test. Differences among multiple groups were examined using a one‐way analysis of variance followed by a Tukey post‐test. Statistical significance was indicated as *p* < 0.05 and expressed as ^*^ *p* < 0.05, ^**^ *p* < 0.01, and ^***^ *p* < 0.001.

## Conflict of Interest

The authors declare no conflict of interest.

## Supporting information

Supporting Information

## Data Availability

The data that support the findings of this study are available on request from the corresponding author. The data are not publicly available due to privacy or ethical restrictions.
