# Supplementary material for: Biofilm Microenvironment Activated Antibiotic Adjuvant for Implant‐Associated Infections by Systematic Iron Metabolism Interference
Source: Adv Sci (Weinh). 2024 Feb 26;11(17):2400862. doi: 10.1002/advs.202400862 (PMC11077648; doi:10.1002/advs.202400862)
Supplement: Supplementary file 1 — Supporting Information [file ADVS-11-2400862-s001.pdf]

ADVANCED SCIENCE
Open Access

Supporting Information

for *Adv. Sci.*, DOI 10.1002/advs.202400862

Biofilm Microenvironment Activated Antibiotic Adjuvant for Implant-Associated Infections by Systematic Iron Metabolism Interference

Jianing Ding, Xin Wang, Wei Liu, Cheng Ding, Jianrong Wu, Renke He* and Xianlong Zhang**

Biofilm Microenvironment Activated Antibiotic Adjuvant for Implant-Associated Infection by Systematic Iron Metabolism Interference

Jianing Ding, Xin Wang, Wei Liu, Cheng Ding, Jianrong Wu, Renke He*, and Xianlong Zhang**

J. Ding, X. Wang, Dr. W. Liu, C. Ding, Dr. R. He, Prof. X. Zhang
Department of Orthopaedics,
Shanghai Sixth People's Hospital Affiliated to Shanghai Jiao Tong University School of Medicine,
Shanghai 200233, P. R. China
E-mail address: sjtuherenke@163.com ; dr_zhangxianlong@sjtu.edu.cn

Dr. J. Wu
Shanghai Institute of Ultrasound in Medicine,
Shanghai Sixth People's Hospital Affiliated to Shanghai Jiao Tong University School of Medicine,
Shanghai 200233, P. R. China
E-mail address: wujianrong028@shsmu.edu.cn

Additional details on experimental section:

Materials: Gallium nitrate hydrate [$Gd(NO_3)_3 \cdot 6H_2O$], aluminium nitrate nonahydrate [$Al(NO_3)_3 \cdot 9H_2O$], magnesium nitrate hexahydrate [$Mg(NO_3)_2 \cdot 6H_2O$], sodium nitrate ($NaNO_3$), N-hydroxysuccinimide (NHS), N-(3-dimethylaminopropyl)-N-ethylcarbodiimide hydrochloride (EDC·HCl), formamide and sodium hydroxide (NaOH) were purchased from the Aladdin Reagent Co. (Shanghai, China). Cefiderocol and deferiprone were obtained from Macklin. PEG-CHO (MW: 3000) was purchased from Tanshtech Co. Ltd (Shanghai, China). Deionized (DI) water (18.2 MΩ·cm) was used in all experiments. The BCA kit, 3,3,5,5-Tetramethylbenzidine(TMB), TMB stopping buffer, DAPI, crystal violet solution, Triton-X-100 solution, bovine serum albumin (BSA), 5(6)-Carboxyfluorescein diacetate succinimidyl ester(CFDA-SE), and 2,7-Dichlorodihydrofluorescein diacetate (DCFH-DA) were purchased from Beyotime Biotechnology (Shanghai, China). The CCK-8 reagent was purchased from Dojindo Molecular Technologies (Kumamoto, Japan). 4% paraformaldehyde was purchased from Wuhan Servicebio Technology Co., (Wuhan, China). Rhodamine-labelled phalloidin was purchased from Yeasen Biotechnology (Shanghai) Co, Ltd. (Shanghai, China). Tryptone soy broth was purchased from Solarbio Science & Technology Co., Ltd. (Beijing, China). LIVE/DEAD BacLight Bacterial Viability Kit, CellTraker Red CMTPX dyes were purchased from Thermo Fisher Scientific (Waltham, MA, USA). Moreover, ELISA kits (TNF-α, IL-1β, MCP-1, IL-10) were purchased from Anogen (Mississauga, Canada). The RNA Purification Kit were purchased from EZBionscience (Roseville, USA). Primers (TNF-α, TNF-β) were purchased from BioTNT (Shanghai, China). Cellular ATP contents were purchased from BacTiter-Glo Microbial Cell Viability Assay (Promega). The TRIzol reagent was purchased from TaKaRa (Kusatsu, Japan). All the other chemicals and reagents were of analytical grade and used as received. Cellular ATP contents were purchased from BacTiter-Glo Microbial Cell Viability Assay (Promega).

Characteristics: TEM and the corresponding energy dispersive spectrum (EDS) mapping were performed on a FEI Talos F200S. XPS analysis was conducted by using ESCALAB 250XI (ThermoFisher, Waltham, MA, USA). The hydrodynamic particle size and zeta potential were determined on a Malvern Zeta sizer Nano series (Nano ZS90, Malvern, UK). The specific surface area of different constructed nanosheets were determined by a Micromeritics Tristar 3000 analyzer (Atlanta, GA). The Ga contents were determined by inductively coupled plasma optical emission spectrometry (ICP-OES, Avio™ 200, PerkinElmer, USA). Laser scanning confocal microscopy (CLSM) were performed on a ZEISS LSM 710. For scanning electron microscopy (SEM), all the samples were performed on a JEOL JSM-7800F. Bacterial oxygen consumption rate (OCR) was quantified on an XFe^{96} Extracellular Flux Analyzer (Agilent).

Spread-plate method (SPM): The bacterial suspensions underwent a tenfold serial dilution, and 100μL of the resulting diluted suspension was spread onto a SBA plate, which was then incubated at 37 °C overnight. The viable bacterial counts were subsequently determined according to the protocol specified in the National Standard of China GB/T 4789.2.

Measurement of metal accumulation: Bacterial cells were harvested at different time points according to the measured OD600. After washing with cold PBS containing EDTA (5 mM), cell pellets were digested with 70% HNO_3 (trace metal basis, Sigma) at 60 °C overnight. Samples were diluted to a final concentration of 5% HNO_3 and analyzed by ICP-MS (iCAP RQ, Thermo Fisher).

The sample processing workflow in transcriptomics: The experiment utilized the TruSeqTM Stranded Total RNA Library Prep Kit to construct the library. During the synthesis of the second strand of cDNA, dUTP was substituted for dTTP in the dNTPs reagent, resulting in the presence of A/U/C/G bases in the second strand of cDNA. Prior to PCR amplification, the second strand was of cDNA was digested using UNG enzyme, ensuring that the library only contained the first strand of cDNA.Total RNA Extraction: Total RNA was extracted from tissue samples. The concentration and purity of the extracted RNA were measured using

Nanodrop2000, and RNA integrity was assessed through agarose gel electrophoresis and Agilent2100 determination of RIN values. For a single library preparation, a minimum of 2 μg of total RNA with a concentration ≥ 100 ng/μL and an OD260/280 ratio between 1.8 and 2.2 was required. rRNA Removal: Unlike eukaryotic mRNA, which possesses a poly A tail at the 3 end, prokaryotic mRNA cannot form A-T base pairs with oligo dT based on poly A. To isolate mRNA from total RNA, rRNA depletion methods are commonly employed, which are used to analyze transcriptome information. mRNA Fragmentation: The Illumina platform is designed for sequencing short sequence fragments. The enriched mRNA obtained is a complete RNA sequence with an average length of several kilobases. Therefore, it needs to be randomly fragmented. By adding fragmentation buffer, mRNA can be randomly broken into small fragments of approximately 200 bp. Reverse Transcription into cDNA: Using reverse transcriptase and random primers, single-stranded cDNA is synthesized from mRNA templates. During the synthesis of the second strand, dUTP is used instead of dTTP in the dNTPs reagent, resulting in the incorporation of A/U/C/G bases in the second strand of cDNA. Adaptor Ligation: The double-stranded cDNA structure has sticky ends, which are repaired to blunt ends by adding End Repair Mix. Subsequently, a terminal A base is added to the 3 end for ligation with Y-shaped adaptors. UNG Enzyme Digestion of cDNA Second Strand: Prior to PCR amplification, the UNG enzyme is used to digest the second strand of cDNA, ensuring that the library only contains the first strand of cDNA. Illumina Hiseq Sequencing: Library enrichment: PCR amplification with 15 cycles. Quantification with TBS380 (Picogreen), followed by pooling the data in the desired proportions for sequencing. Bridge PCR amplification on the cBot instrument to generate clusters. Sequencing on the Illumina Hiseq platform, using 2x150 bp or 2x300 bp sequencing.

Functional Enrichment Analysis: Functional enrichment analysis was performed on the gene set, including Gene Ontology (GO) enrichment and KEGG pathway enrichment. The software Goatools was used for GO enrichment analysis to determine the main GO functions associated

with the genes in the gene set. Fisher's exact test was employed, and a corrected p-value (P adjust) less than 0.05 was considered as significant enrichment of the GO function. For KEGG pathway enrichment analysis, R scripts were used to analyze the genes in the gene set. The principle of calculation is similar to GO enrichment analysis, and a corrected p-value (P adjust) less than 0.05 was considered as significant enrichment of the KEGG pathway function.

GC-MS: The experimental procedure is as follows: Add pre-cooled methanol-water (V:V = 4:1), a total of 1 mL, into the sample, and transfer it to a glass vial in two portions. Add 200μL of chloroform and disperse it using a pipette. Perform ultrasonic disruption in an ice bath at 500 W for 6 minutes with a cycle of 6 seconds on and 4 seconds off. Transfer all the liquid to a centrifuge tube and add 20 μL of internal standard (L-2-chlorophenylalanine, 0.06 mg/mL, prepared in methanol). Perform ultrasonication in an ice-water bath for 20 minutes and let it stand at -40°C for 30 minutes. Centrifuge for 10 minutes (13,000 rpm, 4°C), and transfer a total of 400 μL of the supernatant to a glass derivatization vial for drying. Dry the sample using a centrifugal concentrator. Add 80 μL of methoxy amine hydrochloride pyridine solution (15 mg/mL) to the glass derivatization vial, vortex for 2 minutes, and incubate at 37°C in a shaking incubator for 60 minutes for oximation reaction. After removing the sample, add 50μL of BSTFA derivatization reagent and 20μL of n-hexane to the vial. Add 10 internal standards (C8/C9/C10/C12/C14/C16/C18/C20/C22/C24, all prepared in chloroform) at a volume of 10μL. Vortex for 2 minutes and react at 70 °C for 60 minutes. After removing the sample, let it sit at room temperature for 30 minutes for GC-MS metabolomic analysis.

Evaluation by SEM: In order to investigate the characteristics of adhesive bacteria on the plates, the plates were carefully removed using sterile forceps and washed gently with phosphate-buffered saline (PBS) to eliminate non-adherent bacteria. Next, the adhesive bacteria on the plates were fixed with 2.5% glutaraldehyde at 4 °C for 24 hours after being washed gently three times with PBS. Following this, the bacteria were dehydrated sequentially with a series of alcohol concentrations (50, 70, 80, 90, 95, and 100% v/v) for 10 minutes each in a new 24-well

plate, freeze-dried, coated with platinum, and finally, analyzed using scanning electron microscopy (SEM) with a JEOL JSM-7800F instrument manufactured in Japan.

Evaluation by CLSM: The adhesive bacteria on the plates for each group were transferred to a new 24-well plate, washed with fresh phosphate-buffered saline (PBS), and then stained with a mixture of dyes from the LIVE/DEAD BacLight bacteria viability kit obtained from Invitrogen for 30 minutes. The samples were subsequently imaged using confocal laser scanning microscopy (CLSM). Viable microorganisms were visualized as green fluorescence.

Measurement of ATP: Bacterial cells were harvested after 4 hours of treatment, washed with PBS, and diluted to a concentration of 1×10^8 cells/mL. Next, 100 μL of the bacterial suspension was mixed with 100 μL of BacTiter-Glo reagent and incubated at room temperature for 15 minutes. The luminescence was measured using a BioTek reader. A standard curve was generated using serial dilutions of ATP ranging from 1 μM to 10 pM, and was measured simultaneously with the samples.

Analysis of nascent mRNA transcripts in P. aeruginosa: In the EU labeling experiments, the Click-iT Nascent RNA Capture Kit protocol (Thermo Fisher Scientific) was employed. Bacterial cells at the early log phase were treated with different nanodrugs and pulsed with 0.5 mM 5-ethynyl uridine (EU) for 4 hours. Total RNA was isolated using the SV Total RNA Isolation System (Promega) and subjected to a copper-catalyzed click reaction with azide-modified biotin. The resulting product was then captured on streptavidin magnetic beads that were provided by the kit. cDNA synthesis was performed on the nascent mRNA transcripts captured on the beads directly, using the VILO cDNA synthesis kit (Thermo Fisher Scientific). The obtained product was analyzed by RT-PCR.

Bacterial respiration: The bacterial oxygen consumption rate (OCR) was measured using an XFe96 Extracellular Flux Analyzer. *P. aeruginosa* cultures with an OD600 of approximately 0.01 were seeded onto the assay plate and allowed to attach through centrifugation. DFP@Ga-LDH were loaded into the injection ports, and basal OCR was measured for two cycles before

injection to ensure uniform seeding. After injection, 15 measuring cycles were performed, and each sample was tested in five replicate wells. Data analysis was conducted using Wave Desktop 2.2 software.

Catalase and SOD assays: In this study, *P. aeruginosa* strain PA01 was cultured in Tryptic Soy Broth (TSB) containing various concentrations of nanodrugs. After overnight incubation at 37˚C with shaking, the bacterial cells were harvested, and lysates were generated for the determination of enzyme activity. The protein concentration of the lysates was measured using the bicinchoninic acid (BCA) assay. To determine catalase and superoxide dismutase (SOD) activity, the bacterial pellet was washed in phosphate-buffered saline (PBS) and then lysed using a buffer containing Tris and EDTA, along with lysozyme, either by incubating at 37°C for 4 hours with constant tumbling. The lysate was then centrifuged, and the resulting supernatant was used to measure catalase activity by monitoring the rate of consumption of hydrogen peroxide (H_2O_2) spectrophotometrically. SOD activity was measured using a commercial WST SOD assay kit.

Measuring DFP@Ga-LDH's combined activity with antibiotics: In checkerboard assays, a series of dilutions of the DFP@Ga-LDH nanodrug and antibiotics were mixed in microtiter plates. Each row of the plate contained a fixed amount of DFP@Ga-LDH and increasing concentrations of the antibiotic. Next, 10^6 CFUs of bacteria were added to each well, and the optical density was measured 24 hours later to determine the concentration. The fractional inhibitory concentration (FIC), which is the concentration that inhibits bacterial growth when used in combination divided by the concentration with the same effect when used alone, was then calculated and plotted on an isobologram. Concave isobols indicate synergy between the two compounds, while convex isobols indicate antagonism. In time kill assays, bacterial cultures were grown in media until mid-log phase and diluted to 10^5 CFUs/mL. DFP@Ga-LDH, antibiotic, or a combination of both were added at the specified concentrations at time zero, and samples were taken at different time points for viable CFUs measurement.

Oxidant sensitivity assays: *P. aeruginosa* bacterial cultures were prepared at a concentration of 10^6 CFUs per milliliter in new plastic containers. The cultures were supplemented with the DFP@Ga-LDH and PBS and then grown with shaking in polystyrene tubes for 24 hours. Approximately 10^7 CFUs were collected and subjected to oxidative stress by exposure to different concentrations of H_2O_2, tert-butyl for one hour. The surviving CFUs were then quantified by dilution plating.

Histological Observation: The soft tissue samples surrounding the implants from each group were collected and fixed in 10% buffered formalin at 4°C for 3 days. After being washed with fresh PBS, they were dehydrated in a graded series of alcohol solutions and embedded in paraffin. The samples were then sectioned using a sledge microtome from Leica, Hamburg, Germany. Finally, the sections were deparaffinized in xylene. For histological analysis, Hematoxylin and Eosin (H&E) staining and Giemsa staining were performed, and the resulting changes were visualized using an optical microscope.

Immunofluorescent Staining of biomarkers in the tissues: Paraffin-embedded soft tissue sections were stained with primary antibodies, incubated with secondary antibodies and corresponding fluorescent markers in batches. The primary antibodies: rabbit anti-mice MPO (servicebio), rabbit anti-mice F-480 (servicebio). The secondary antibodies: goat anti- rabbit general secondary antibodies. The fluorescent markers: CY3 agent, FITC agent, CY5 agent. Finally, the sections were stained with DAPI. All images were captured on a Nikon fluorescent microscope equipped with a digital camera (TE2000-U, Nikon). Each immunofluorescent stain was repeated three times using serial sections. Negative controls were included to determine the amount of background staining.

Microbiological Evaluation in vivo: To quantitatively evaluate the bacterial load *in vivo*, subcutaneous PEEK plates from each group were aseptically removed and washed twice with PBS to remove the planktonic bacteria. The plates were then immersed in 1mL of PBS, sonicated for 10 min at 150 kHz, and vortexed for 2 min to dissociate the adherent bacteria.

Soft tissues surrounding the implants were also obtained, weighed, and homogenized in sterile tubes with 1 mL of PBS using a high-speed homogenizer (Jingxin Industrial Limited Company, Shanghai, China). The homogenates of soft tissue were then serially diluted with PBS, and viable bacteria from the dilutions were counted using the SPM.

Cytotoxicity measurements of DFP@Ga-LDH-Cefi: Rat bone marrow mesenchymal stem cells (rBMSCs) and mouse embryonic fibroblasts (NIH3T3) were selected to assess the potential cytotoxicity of DFP@Ga-LDH-Cefi. In brief, these cells were separately seeded in a 96-well microplate at a density of 1×10^4 cells per well (in 100 μL corresponding culture medium) and allowed to overnight culture. The culture medium was then replaced with 100 μL of fresh medium containing varying concentrations of DFP@Ga-LDH-Cefi. After further incubation for 24 hours, the culture medium was removed and replaced with fresh medium containing 10% CCK-8, followed by an additional 3-hour incubation. 100 μL of the incubated culture medium from each group was transferred to a new 96-well plate, and the absorbance was measured at 450 nm using a microplate reader.

Quantification of biomarkers for inflammatory cells: Neutrophil infiltration and macrophage recruitment were assessed by quantifying myeloperoxidase (MPO) and β-N-Acetylglucosaminidase (NAG) levels, respectively. Soft tissue samples around the implants were collected, weighed, and homogenized, and MPO and NAG levels were measured using commercially available assay kits (Sigma-Aldrich) following the instructions of manufacturer.

Hemolysis Analysis of nanodrugs: Hemolysis analysis was performed on red blood cells (RBCs). RBCs were collected by centrifugation at 1500 rpm for 15 minutes and washed five times with saline. The centrifuged RBCs (2.5 mL) were mixed with saline (10 mL) to prepare a stock suspension. Then, 100 μL of the stock suspension was added to 1 mL of DFP@Ga-LDH-Cefi dispersions with different concentrations. The final hematocrit level of RBCs was approximately 4%. The solutions were incubated at 37 °C for 4 hours. After centrifugation at 12000 rpm for 15 minutes, the percentage of hemolysis was measured by UV-Vis analysis of

the supernatant at 540 nm absorbance. Saline and pure water were used as negative and positive controls, respectively. All samples were prepared in triplicates. The hemolysis percentage was calculated using the following formula: Hemolysis (%) = (C-A) / (C-B) × 100%, where A is the absorbance of DFP@Ga-LDH-Cefi with RBCs suspension, B is the absorbance of saline, and C is the absorbance after the addition of deionized water.

Toxicity measurements of DFP@Ga-LDH-Cefi in vivo: The animal experiments for this study were approved by the Animal Care and Experiment Committee of the Shanghai Jiao Tong University affiliated Sixth People's Hospital. Eighteen female ICR mice at 7 weeks (Shanghai Laboratory Animal Center, Chinese Academy of Sciences) were chosen for *in vivo* toxicity measurement. The mice were intravenously injected with a dosage of 40 mg/kg DFP@Ga-LDH-Cefi. We measured the body weights of the mice every three days, with the same volume of saline used as the control group. After 28 days, the mice were sacrificed, and their major visceral organs (heart, liver, spleen, lung, and kidney) were collected. The other major visceral organs were preserved in a 10% formalin solution for further histopathology analysis using a typical hematoxylin and eosin (H&E) staining assay to further evaluate potential *in vivo* toxicity. The complete blood panel parameters, including aspartate aminotransferase (AST) and alanine aminotransferase (ALT), were measured in all groups using authoritative standard biochemistry tests.

Measurement of hematoma-created iron concentration: Titanium rod (the length is 15mm and the diameter is 1mm) was inserted into right femur of 12-week-old Sprague-Dawley rat. The procedure was conducted as described in our previous report (G. Guo, *Advanced Functional Materials* **2021**, 31) Following the implantation, on days 1,7,14, and 28, animals are sacrificed and the right femur and associated soft tissue are taken. Tissues are weighted and iron concentration are measured by inductively coupled plasma optical emission spectroscopy (ICP-OES).

Macrophage assays: Peripheral blood samples were collected from healthy volunteers who provided written consent. The study was approved by the Ethics Committee of Shanghai Sixth People Hospital and conducted in accordance with relevant regulations and institutional guidelines. The approval number assigned to this is 2022-KY-139(K). Peripheral blood monocytes from healthy individuals were isolated and differentiated into human monocyte-derived macrophages (HMDM) using M-CSF treatment. Human monocyte-derived macrophages (HMDM) were exposed to either a control vehicle or DFP@Ga-LDH at a concentration of 1 mg/ml for a duration of 24 hours. The samples were treated with either Control or DFP@Ga-LDH. Viability of HMDM was evaluated using calcein-AM staining, while gene expression was analysed through qRT-PCR. To assess bacterial killing, HMDM treated with control or DFP@Ga-LDH were incubated with *P. aeruginosa* (PA01) to facilitate phagocytosis. HMDM were treated with gentamicin to eliminate non-internalized bacteria. Subsequently, the cells were incubated for different durations (0h, 2h, 3h), lysed, and the live bacteria were quantified by plating them on agar to determine the number of colony forming units.

*Ga concentrations in the major visceral organs***:** To conduct *in vivo* toxicity measurements, female ICR mice aged 7 weeks were selected from the Shanghai Laboratory Animal Centre, Chinese Academy of Sciences. Mice were administered DFP@Ga-LDH (40 mg/kg) via intravenous (*i.v.*) injection, while the control group received an equivalent volume of saline. Mice were sacrificed at three time points: 0 days prior to intravenous injection, 7 days after injection, and 28 days after injection. The major visceral organs (heart, liver, spleen, lung, and kidney) were collected for analysis. The concentration of Ga in major visceral organs was determined using inductively coupled plasma optical emission spectroscopy (ICP-OES) following the dissolution of the organs.

Morphology of treated P. aeruginosa: TEM was used to determine the attachment of DFP@Ga-LDH to the bacteria. After a 6-hour co-culture of *P.aeruginosa* (at a concentration of 10^6

CFUs/mL) with DFP@Ga-LDH (at a concentration of 20 mg/mL), the bacterial cells were collected and washed using cold phosphate-buffered saline (PBS) containing EDTA (5 Mm) . The cell pellets were fixed using a 2.5% glutaraldehyde solution for a duration of 3 hours, followed by three washes with PBS. The bacteria were subsequently fixed by immersing them in a 1% aqueous solution of OsO4 at room temperature for a duration of 2 hours. The samples were washed three times with PBS and dehydrated using a series of ethanol solutions (30%, 50%, 70%, 80%, 90%, 100%) for 15 minutes each. Subsequently, they were permeated with a medium consisting of acetone and EPON 812 in a 1:1 ratio, followed by pure EPON 812 for a duration of 12 hours. Subsequently, the samples were subjected to embedding at a temperature of 60 °C for a duration of 48 hours. Following this, the samples were transformed into sheets measuring 60 nm using a microtome (Leica UC7) fitted with a diamond knife (Tecnai G2 20 TWIN). Finally, the sheets were stained with uranylacetate. TEM was used to observe the section structure of *P. aeruginosa*.

Table S1. The structural parameters of different constructed nanosheets.

**Formulation**	**BET Surface area (m^2/g)**	**Hydrodynamic size (nm)**	**PDI**	**Zeta potential (mV)**
Ga-LDH	267.4	113.3 ± 3.5	0.23 ± 0.09	39.7 ± 1.5
DFP@Ga-LDH	178.5	119.6 ± 2.8	0.27 ± 0.06	18.7 ± 2.7
DFP@Ga-LDH-Cefi	116.5	120.6 ± 3.3	0.33 ± 0.05	6.5 ± 3.3

Supplementary Figures

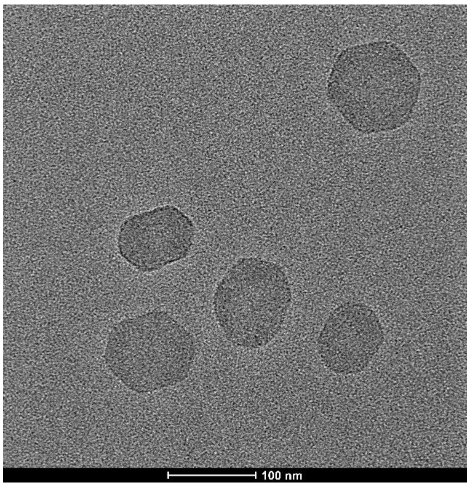

Figure S1. TEM image of DFP@Ga-LDH-Cefi.

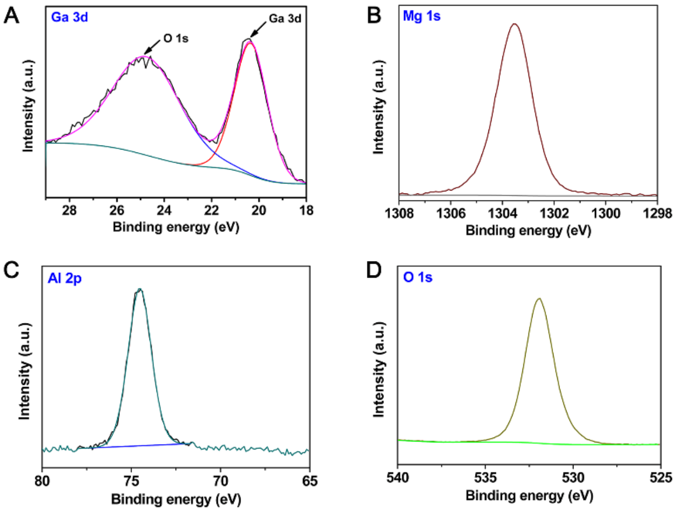

Figure S2. XPS data showing the (A) Ga 3d, (B) Mg 1s, (C) Al 2p, and (D) O 1s spectrum.

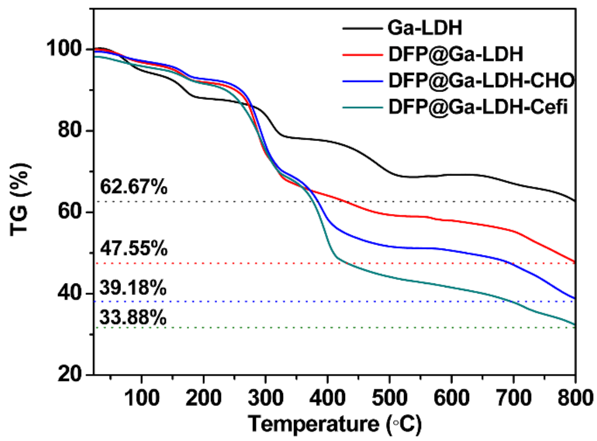

Figure S3. TGA curves of Ga-LDH, DFP@Ga-LDH, DFP@Ga-LDH-CHO, and DFP@Ga-LDH-Cefi.

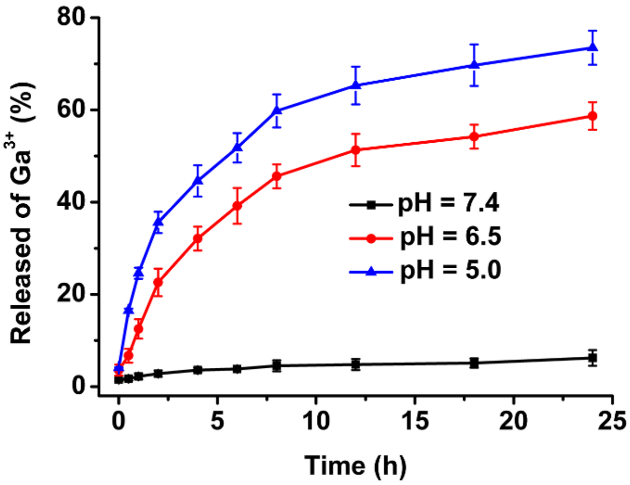

Figure S4. The release profile of Ga^{3+} from DFP@Ga-LDH-Cefi in PBS at different pH values.

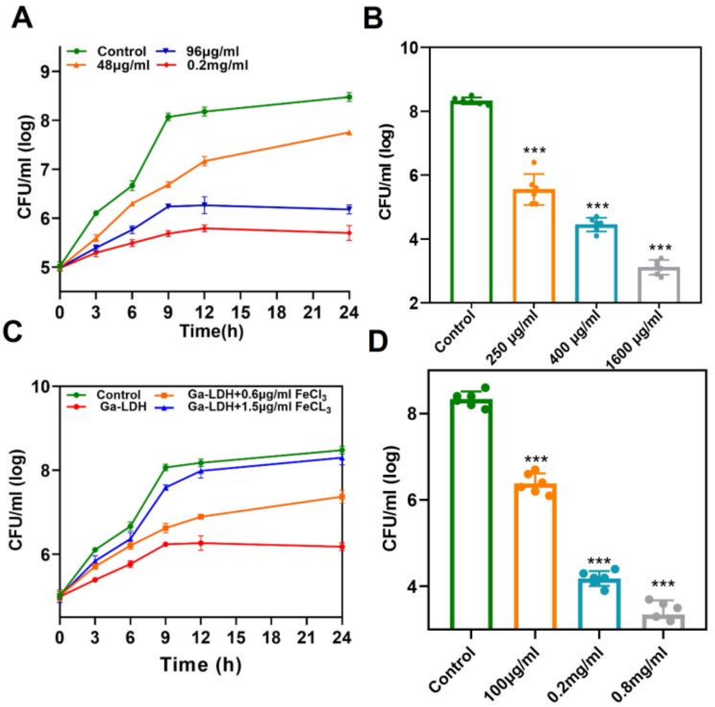

Figure S5. **(A)** Effect of Ga-LDH on the growth of *P. aeruginosa*. Results are representative of six. **(B)** 24-h bactericidal assay of Ga-LDH nanosheets against stationary phase *P. aeruginosa*. **(C)** The addition of ferric chloride weakened the antibacterial efficiency of Ga-LDH. **(D)** 24-h bactericidal assay of DFP@Ga-LDH nanosheets against stationary phase *P. aeruginosa*. (**P$<$0.01, ***P$<$0.001)

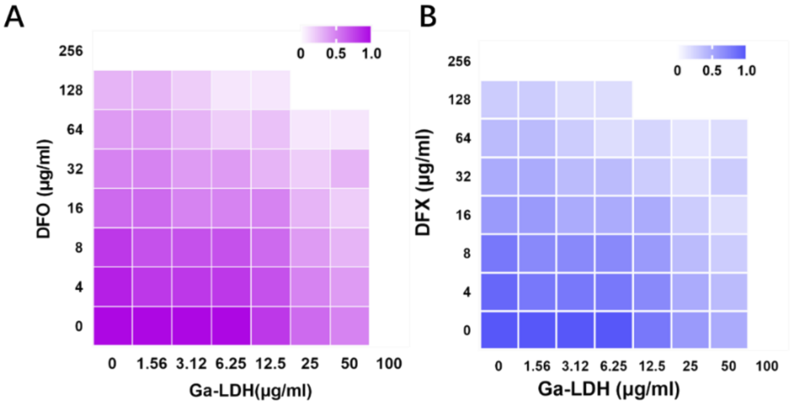

Figure S6. Representative heat plot of checkerboard microdilution assay for the combination of Ga-LDH and DFO, DFX against *P. aeruginosa*.

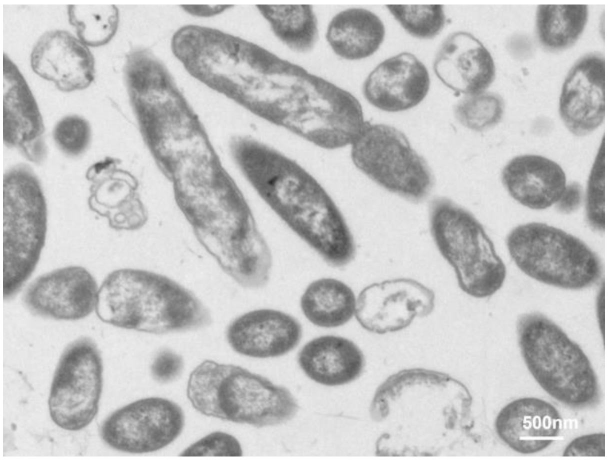

Figure S7. TEM performed on *P. aeruginosa* samples that were co-cultured with DFP@Ga-LDH for a duration of 6 hours.

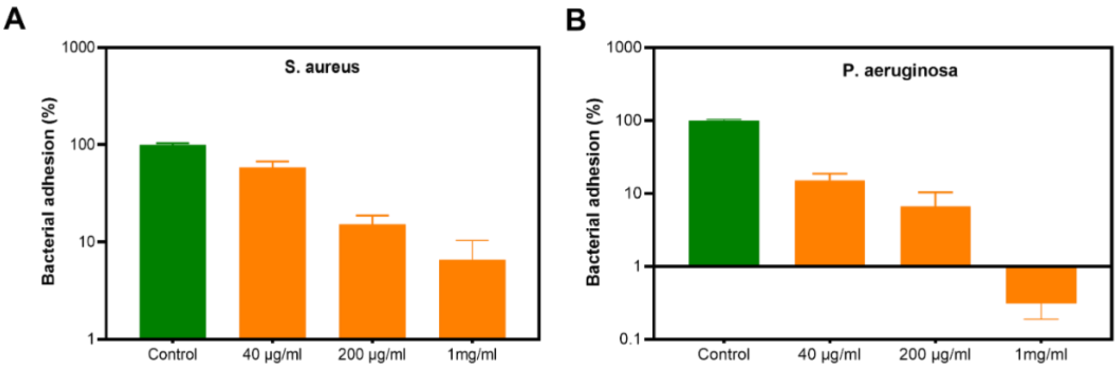

Figure S8. Bacterial adhesion inhibition of *P. aeruginosa* and *S. aureus* after supplemented with DFP@Ga-LDH with various concentration.

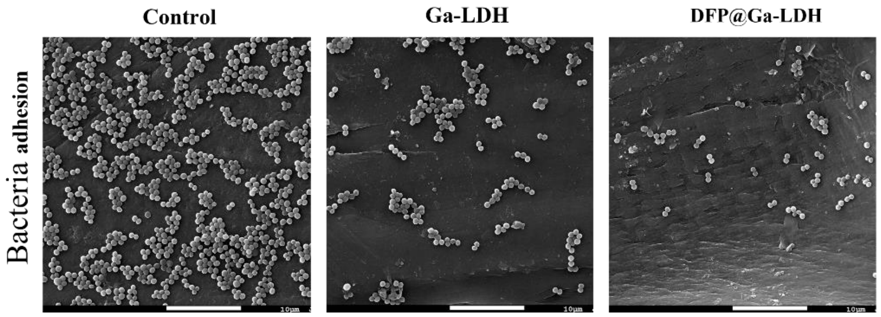

Figure S9. SEM images indicating bacterial adhesion of *P. aeruginosa* by DFP@Ga-LDH on Peek plate.

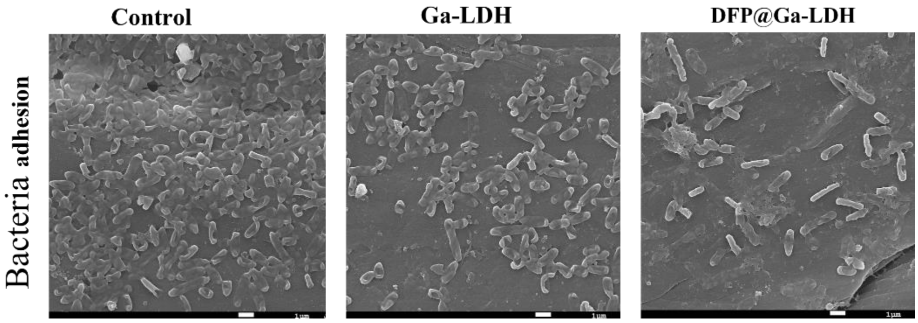

Figure S10. SEM images indicating bacterial adhesion of *S.aureus* by DFP@Ga-LDH on Peek plate.

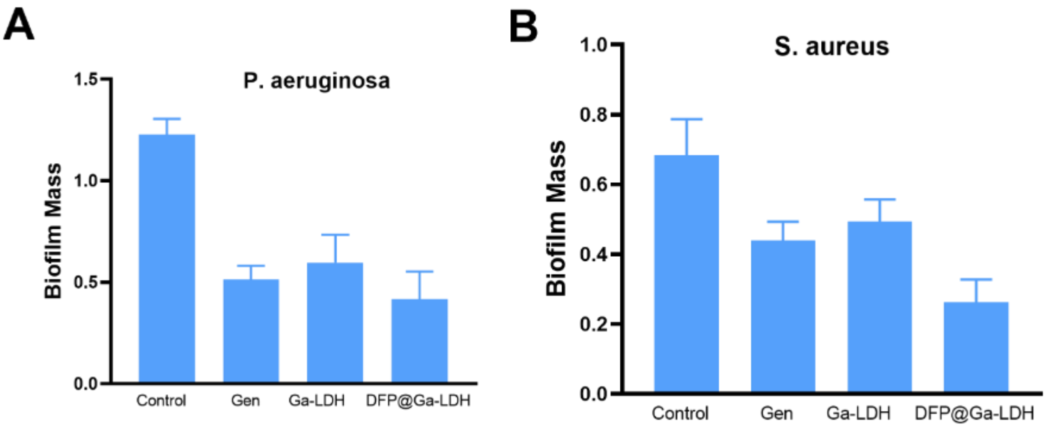

Figure S11. Biofilm formation assessment of *P.aeruginosa* **(A)** and *S.aureus* **(B)** after supplemented with gentamysin, Ga-LDH and DFP@Ga-LDH.

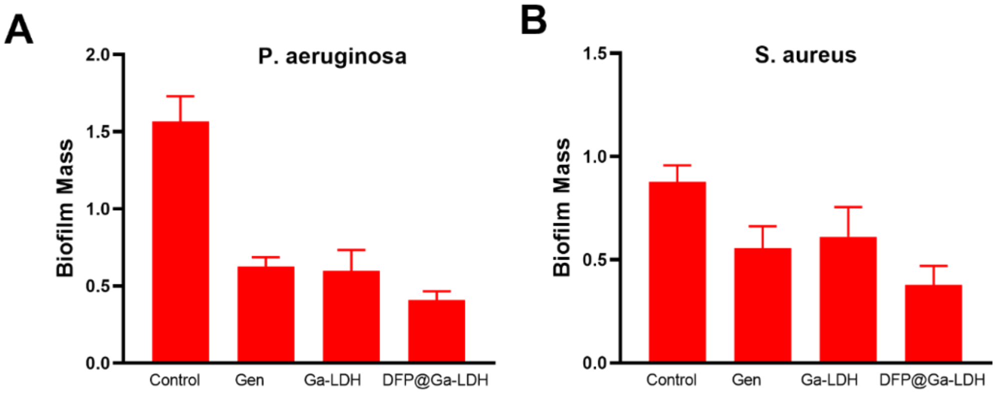

Figure S12. Eradication of established biofilm of *P. aeruginosa* **(A)** and *S. aureus* **(B)** after supplemented with gentamysin, Ga-LDH and DFP@Ga-LDH.

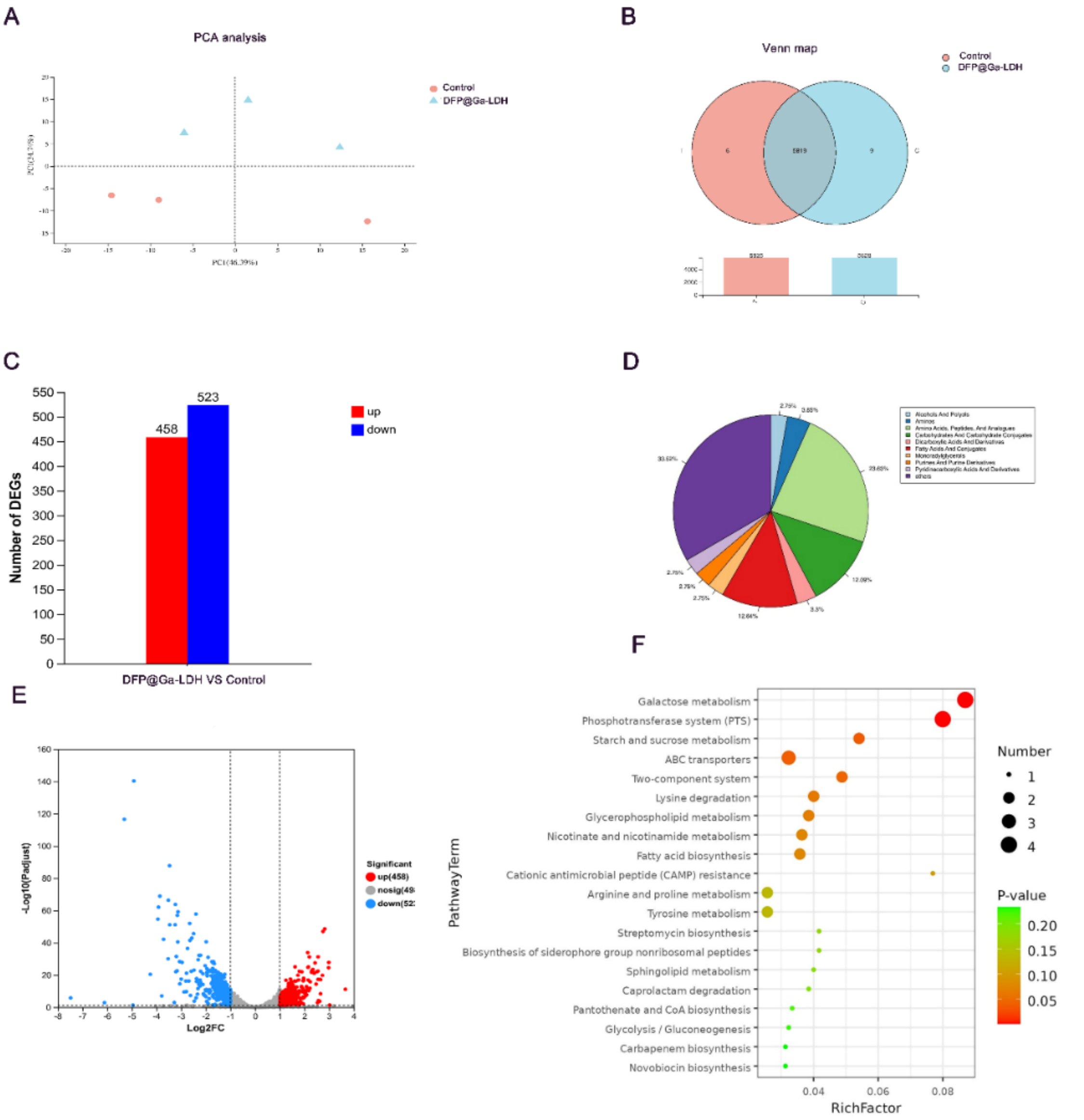

Figure S13. (A) PCA Analysis of the transcriptomics. **(B)** Venn analysis of transcriptomics. **(C)** Differential expression statistical bar chart of transcriptome. **(D)** Pie-Class of metabolomics. **(E)** Volcano plot of metabolomics. **(F)** TOP-20 bubble chart of metabolism.

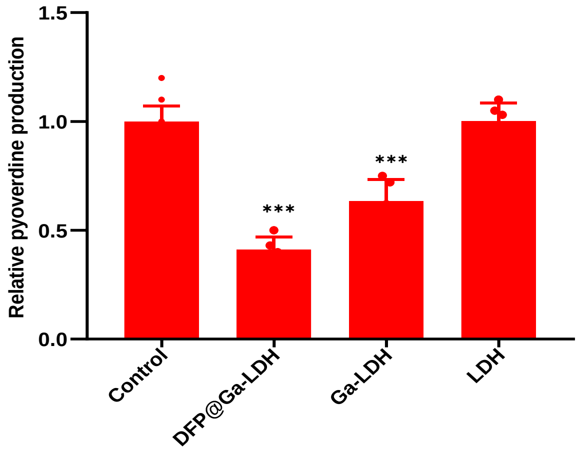

Figure S14. Inhibition of Pyoverdine production after supplemented by DFP@Ga-LDH and Ga-LDH. (***,P＜0.001)

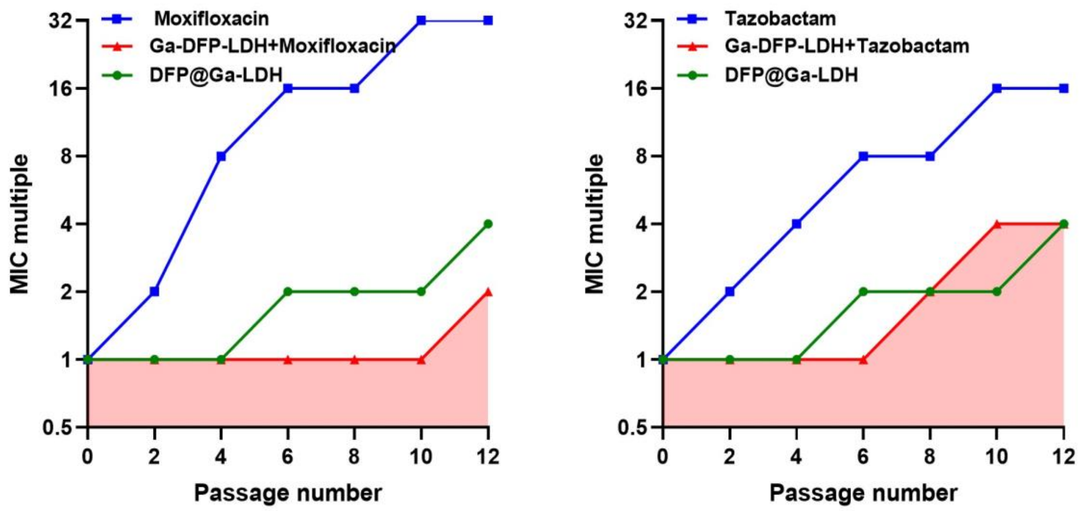

Figure S15. Resistance acquisition curves with antibiotics and DFP@Ga-LDH against *P. aeruginosa*.

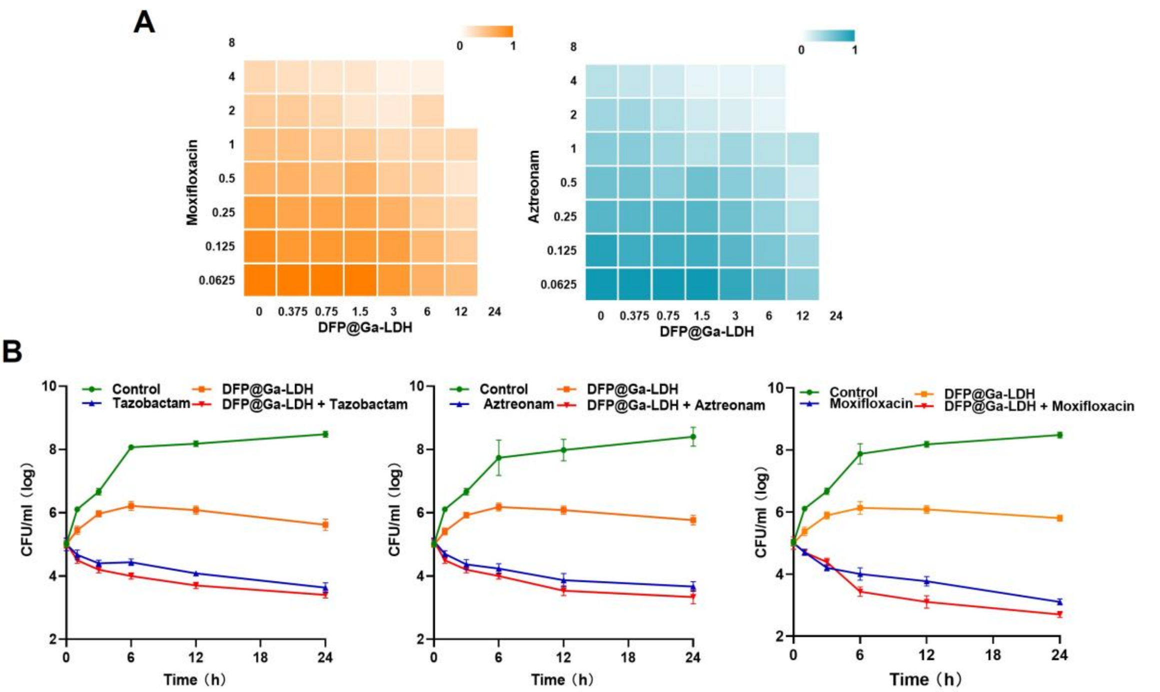

Figure S16. (A) Checkerboard microdilution assay for the combinations between DFP@Ga-LDH and moxifloxacin, aztreonam. **(B)** The synergistic effects between DFP@Ga-LDH and tazobactam, moxifloxacin and aztreonam.

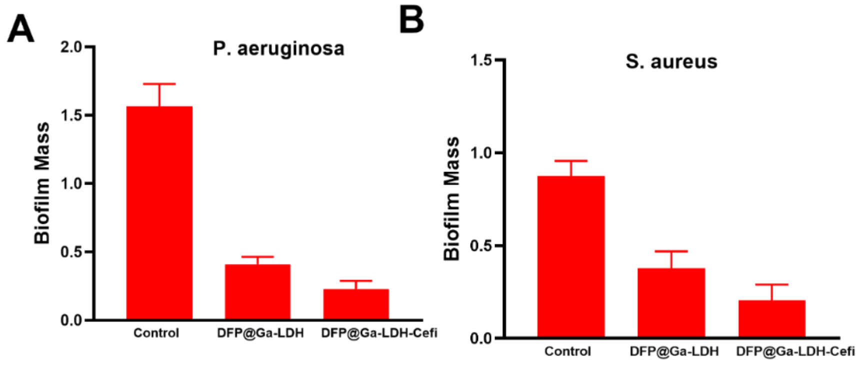

Figure S17. Eradication of established biofilm of *P. aeruginosa* **(A)** and *S. aureus* **(B)** after supplemented with DDP@Ga-LDH and DFP@Ga-LDH-Cefi.

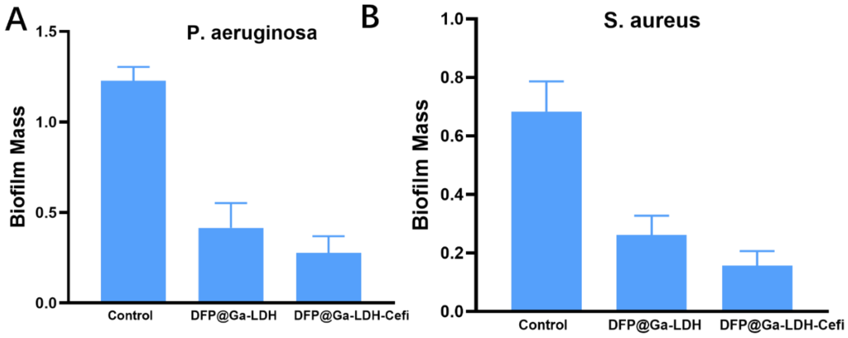

Figure S18. Biofilm formation assessment of *P.aeruginosa* **(A)** and *S.aureus* **(B)** after supplemented with DFP@Ga-LDH and DFP@Ga-LDH-Cefi.

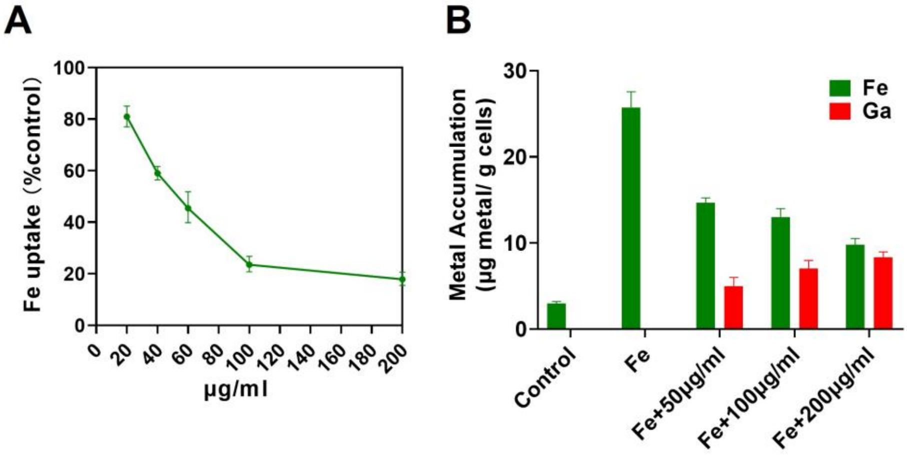

Figure S19. (A) Iron clearance by DFP@Ga-LDH-cefi. **(B)** Metal accumulation of *P. aeruginosa* under different Fe and DFP@Ga-LDH-Cefi treatment.

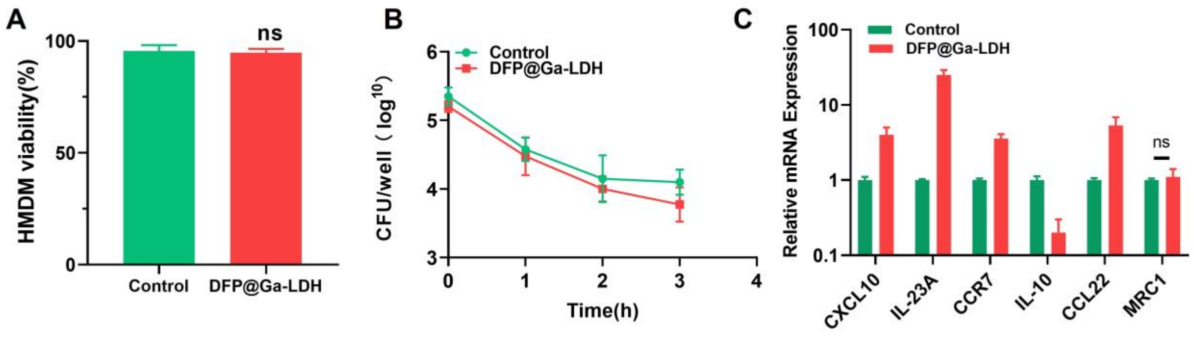

Figure S20. HMDM were treated with vehicle, or DFP@Ga-LDH (1 mg/ml) for 24h. **(A)** The viability of HMDM. **(B)** *P. aeruginosa* killing by HMDM at various time points. **(C)** Relative mRNA abundance of transcripts involved in polarization.

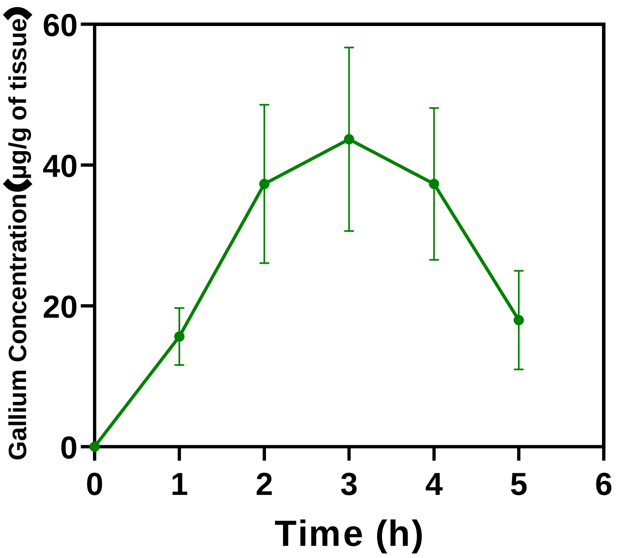

Figure S21. Quantification of the gallium concentration at the infected tissues at various time points following implantation.

group.

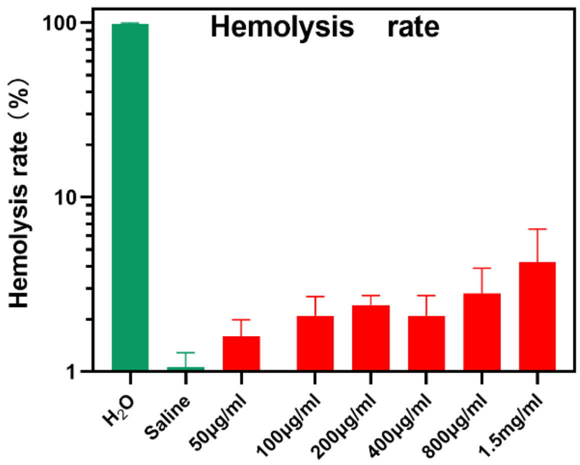

Figure S22. Hemolysis rate of RBC after incubation with DFP@Ga-LDH-Cefi.

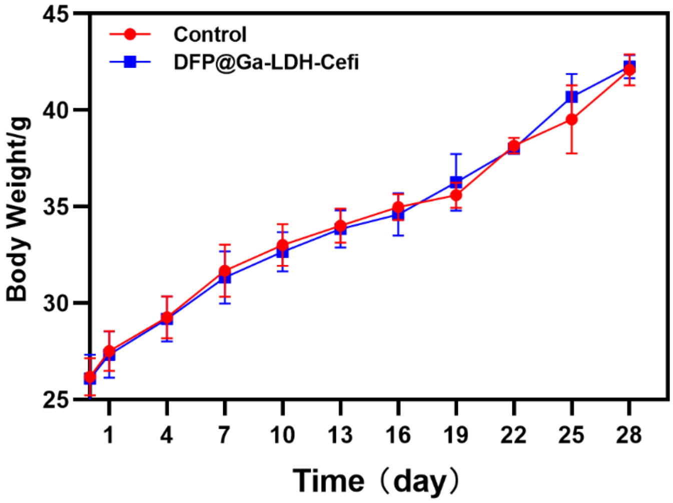

Figure S23. Mice weight measurements of the control group and DFP@Ga-LDH-Cefi treated group.

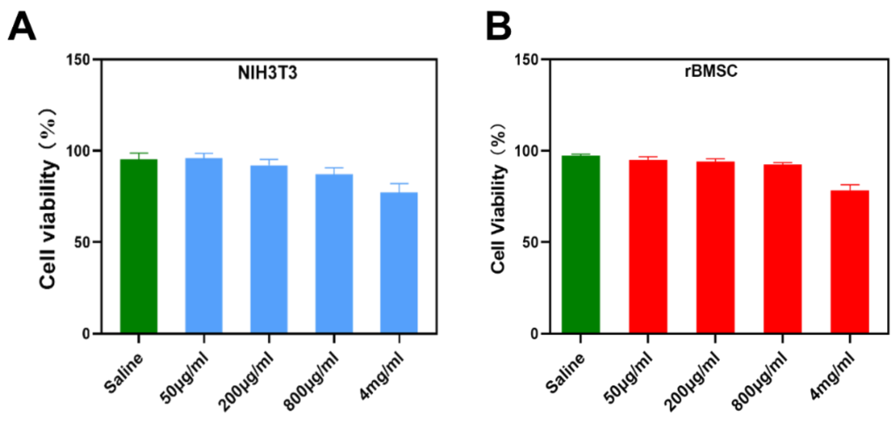

Figure S24. Cell viabilities of rBMSC and NIH3T3 cells after incubation with DFP@Ga-LDH-Cefi for 24h.

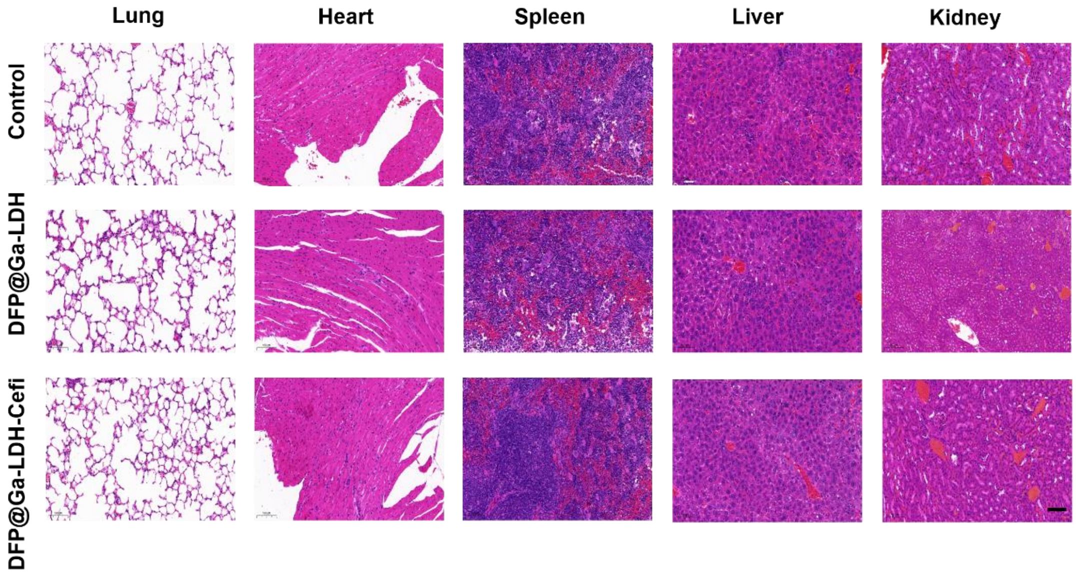

Figure S25. H&E histological images of major organs (heart, liver, spleen, lung, and kidney) from mice after different treatments. (Scale bar = 100 μm)

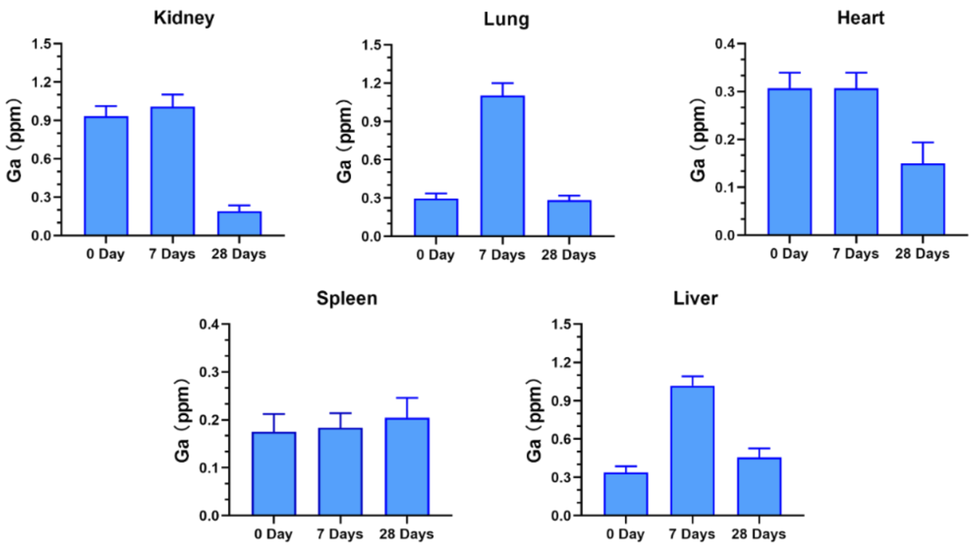

Figure S26. Ga concentrations in the major visceral organs of mice measured by ICP-OES. The major visceral organs collected at three time points (0, 7, and 28 days) following the intravenous DFP@Ga-LDH administration (4 mg/100g).

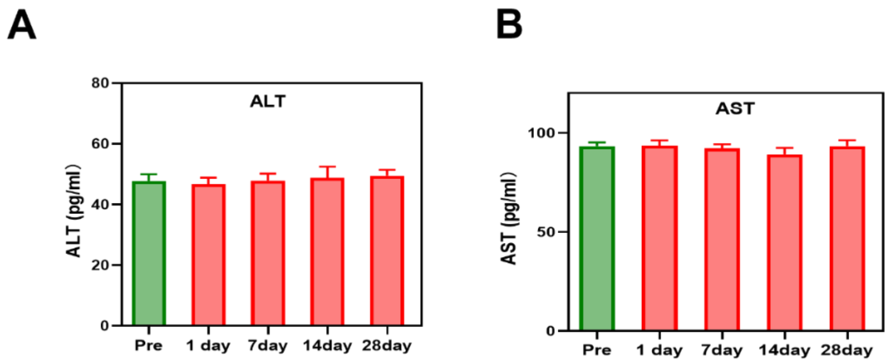

Figure S27. Blood biochemical parameters obtained from the mice after the intravenous injection of DFP@Ga-LDH-Cefi in different times.

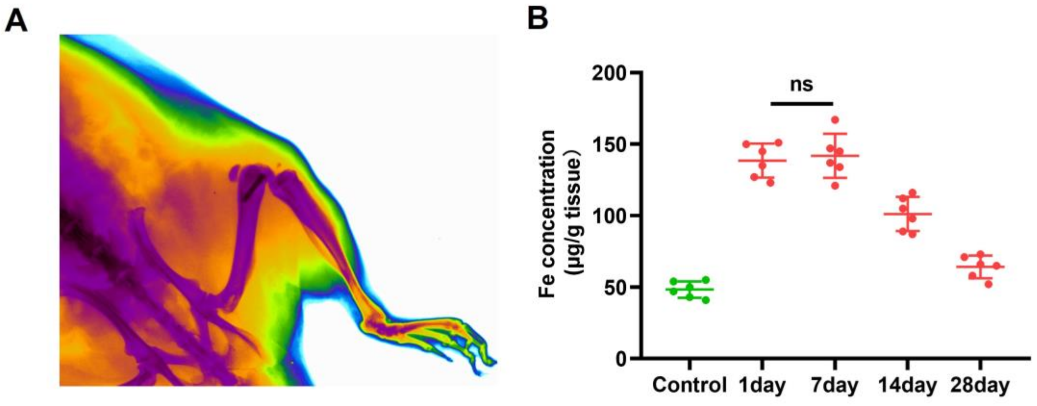

Figure S28. (A) X-ray performed immediately after titanium rod implantation. **(B)** Fe concentration within tissues around implant (ns, not significant).
